# Applications and limitations of the passenger hypothesis for HIV reservoir persistence and cure

**DOI:** 10.1128/jvi.00714-25

**Published:** 2025-06-04

**Authors:** Joshua T. Schiffer, Joseph C. Mudd, Annukka A. R. Antar, Adam M. Spivak, Daniel B. Reeves

**Affiliations:** 1 Vaccine and Infectious Diseases Division, Fred Hutchinson Cancer Center 561181 , Seattle, Washington, USA; 2 Clinical Research Division, Fred Hutchinson Cancer Center 7286, Seattle, Washington, USA; 3 Department of Medicine, University of Washington 205280, Seattle, Washington, USA; 4 Tulane University National Primate Center, Covington, Louisiana, USA; 5 Department of Microbiology and Immunology, Tulane University School of Medicine 12255, New Orleans, Louisiana, USA; 6 Department of Medicine, Johns Hopkins University 229384, Baltimore, Maryland, USA; 7 Department of Medicine, University of Utah, Salt Lake City, Utah, USA; 8 Department of Global Health, University of Washington 150331, Seattle, Washington, USA; New York University Department of Microbiology, New York, New York, USA

**Keywords:** human immunodeficiency virus, mathematical modeling

## Abstract

Antiretroviral therapy (ART) suppresses HIV replication in people living with HIV (PWH), but a persistent population of reservoir cells prevents cure. Reservoir cells are mostly anatomically dispersed, latently infected CD4+ T cells harboring one copy of chromosomally integrated, replication-competent HIV proviral DNA. Despite their low frequency (0.01%–0.1%) among CD4+ T cells and the quiescence of most genetically intact proviruses, viremia usually recurs within weeks after ART cessation. When PWH are not on ART, the reservoir is sustained through viral infection and infected cell proliferation. During suppressive ART, HIV reservoir cells persist via mechanisms sustaining uninfected CD4+ T cells including antigen-responsive and homeostatic clonal proliferation, programmed cell death, and T cell subset differentiation. Rates of latently infected cell proliferation and death must exist in quasi-equilibrium to explain limited change in reservoir volume over decades of ART, and the rarity of cancers or lymphoproliferative disorders emerging from infected cells. Some reservoir cells are under additional selection forces during ART, illustrated by slightly higher clearance rates of genetically intact versus replication-defective HIV proviral DNA and by a gradual transition to a less transcriptionally active and more clonal reservoir. While a small but meaningful percentage of latently infected cells are negatively selected due to lytic viral replication or elimination by adaptive immune responses, most reservoir cell death occurs independently of harboring intact HIV DNA. Given that HIV is often a passenger in reservoir cells, CD4+ T cell proliferation, targeted death, and subset differentiation may be viable therapeutic targets for curative interventions.

## THE PASSENGER HYPOTHESIS

The goal of this review is to summarize evidence supporting and deviating from the hypothesis that chromosomally integrated HIV DNA is a relatively quiescent passenger within latently infected CD4+ T cells in people living with HIV (PWH) on long-term suppressive antiretroviral therapy (ART). The passenger hypothesis explains key observations of reservoir cell dynamics including clonal proliferation, programmed cell death, and subset differentiation of latently infected CD4+ T cells but is not absolute, as genomically intact and some near-intact HIV proviruses are subject to negative selection due to cytolytic effects of the virus or immune recognition. However, the natural dynamic behaviors of uninfected CD4+ T cells are fundamental to HIV reservoir persistence and are necessary to consider for optimizing cure strategies. While the relative importance of proliferation, death, subset differentiation, and selection shifts according to stage of HIV infection and host immune status, the passenger hypothesis is critical for explaining a substantial proportion of reservoir dynamics during untreated and treated HIV.

## THE HIV RESERVOIR

In this review, we define the HIV reservoir as the population of latently infected cells which harbor inducible, replication-competent HIV proviruses that can initiate viral rebound if ART is stopped in PWH. The reservoir persists at clinically meaningful levels over decades of ART with very slow decay during the first ~7 years of ART followed by stabilization (1–4). A majority of reservoir cells are CD4+ T cells, which traffic between blood, lymphoid tissues, and peripheral organs (5). The number of reservoir cells in most PWH on ART is estimated to range between 1 and 100 million (3). When ART is stopped, rebound viremia usually occurs within 1–4 weeks at levels similar to those prior to ART initiation (6–8). Manifestations of untreated HIV reappear, including CD4+ T cell depletion, progression to AIDS, and death (9, 10). Because halting viral replication does not eliminate the reservoir, most PWH must remain on ART for their entire life.

## LATENTLY INFECTED CD4+ T CELLS

During untreated HIV, both actively infected and latently infected CD4+ T cells are present and are distinguished by several attributes (11). Actively infected CD4+ T cells are transcriptionally activated and support rapid amplification of HIV RNA and HIV protein expression on the cell surface, leading to cell lysis after approximately a day as well as potential targeting by immune cells (12–15). In latently infected cells, the viral replication cycle stops after reverse transcription of HIV RNA and integration of HIV DNA into the human chromosome. Latently infected CD4+ T cells are in a resting state, which allows the cell to survive for a much longer time (16, 17). During untreated infection, only a small proportion of newly infected CD4+ T cells enter latency. It is hypothesized that these cells may be in the process of reverting from an activated to quiescent state, though potential mechanisms giving rise to latently infected cells may be multifactorial (18). Measuring the reservoir during untreated HIV is nuanced because a significant fraction of HIV DNA is not chromosomally integrated and exists as unstable linear forms of viral DNA or quasi-stable 2-long terminal repeat (LTR) circles (19–21).

A remarkable feature of latently infected cells is that in blood and lymphatic tissue, they usually only contain a single HIV DNA molecule integrated into human chromosomal HIV DNA (22–24). The quantity of proviral molecules can therefore be used as a convenient surrogate for the number of latently infected cells during sustained ART. Exclusion of multiple proviral integrations is likely due to HIV accessory protein-induced recall of CD4 molecules from the cell surface followed by proteasomal degradation (25–28). HIV accessory proteins also counter cellular restriction factors and contribute to immune evasion during active viral replication, though their role in the establishment and maintenance of latency remains unclear (29–31). While integration somewhat preferentially occurs in cellular growth genes and near Alu repeats which tend to cluster near active cellular genes, each integration site is unique, and therefore, integration sites can be used as a natural barcode (16, 32–34).

## QUANTIFICATION OF THE HIV RESERVOIR

The HIV reservoir can be quantified using several different assays, each with relative strengths and limitations. The most sensitive approach is total HIV DNA using qPCR of one genomic location (e.g., LTR or *gag*). A critical drawback is that 90%–98% of HIV DNA sequences contain insertions, deletions, or mutations rendering them replication incompetent. The percentage of replication-incompetent proviruses also tends to increase with time on ART (1, 35–39).

Quantitative viral outgrowth assays (QVOAs) are the gold standard for measuring inducible proviral levels. QVOA involves serial dilution of CD4+ T cells to identify single cells which generate productive viral replication following one or more rounds of supraphysiologic stimulation with anti-CD3/CD28 antibodies or phorbol 12-myristate 13-acetate (PMA) (2–4, 40, 41). QVOA is labor-intensive, requires a large input of cells/large blood draw, takes weeks, and one round of stimulation fails to induce 90%–99% of replication-competent proviruses (1). In cure trials, participants with small reservoirs often enter with undetectable viral outgrowth (42). As such, QVOA is vital for understanding reservoir dynamics but has limited use as a trial endpoint.

In recent years, elegant multi-probe assays have emerged as a high-throughput, precise, and reliable method to balance the sensitivity of HIV DNA with the specificity of QVOA (38, 39, 43). Nevertheless, several issues must be considered when interpreting intact HIV DNA results. First, observed ratios of QVOA to intact HIV DNA and intact to total HIV DNA are highly variable among PWH on ART (1). This variability reflects inter-host differences in viral and immune characteristics rather than assay performance, but still complicates endpoint selection and statistical analysis in therapeutic intervention trials. Second, intact HIV DNA levels are sometimes undetectable in PWH with small reservoirs who initiate ART early during infection, but these individuals may still have rebound viremia after analytical treatment interruption (ATI) (8, 44). This issue relates not to assay quality but rather to unavoidable undersampling of all relevant lymphatic tissues where the majority of latently infected CD4+ T cells reside (45). PCR assays used to quantify the burden of hematologic malignancies share this problem: molecular remission following chemotherapy is often followed by relapse (46). Finally, imperfect specificity remains at least a theoretical issue, as even 5% misclassification of defective viruses as intact might lead to underestimation of a promising therapeutic effect if intact HIV DNA is selectively depleted (47). Updated assays employing four or five PCR probes enhance specificity, but at the cost of sensitivity, leading to smaller reservoir size estimates (39, 48). Given these nuances, reservoir assays must always be carefully selected to address the specific scientific questions of a given study (49).

## LONG-TERM STABILITY OF HIV PROVIRAL LEVELS AND THE PASSENGER HYPOTHESIS

During the first year of ART, significant decay of intact HIV DNA is observed, reflecting that many remaining HIV-infected cells are not in a state of latency (50). Yet, in the hundreds of PWH studied to date who have been on suppressive ART for at least a year, the reservoir is generally stable over short time intervals. Spontaneous and sustained rapid and extensive expansion or contraction of the HIV reservoir is rarely observed (2, 3, 35, 41, 51–53). This broadly implies that HIV provirus does not impact the relative birth and death rates of most latently infected cells.

Total HIV DNA, intact HIV DNA, and QVOA have slight but critically important differences in clearance kinetics within individuals on sustained ART. Total HIV DNA levels (which include mostly defective and some intact proviruses) remain largely unchanged after 5 years on ART after a median rate of decline of 0.05 logs/year during years 1–5 of treatment (52–54). In a cohort of 1,057 individuals on ART with a minimum of three sequential samples, it was extremely rare for total HIV DNA levels to decrease or increase by more than 0.5 log between years 1.5 and 5.4 of ART (53). Intact HIV DNA levels undergo multi-phasic decay during sustained ART with rapid clearance (half-life = 12.9 days) during the first several months, slow clearance (median half-life 19 months) over the next 1–2 years (50), extremely slow clearance through 7 years of ART (median half-life 4 years) (39, 54), and stabilization thereafter (51, 54). QVOA-outgrowth proviruses decrease with a median half-life of 44 months during the first 7 years of sustained ART (2, 41, 51), while new evidence suggests that levels of outgrowth-competent HIV stabilize and may even increase extremely slowly after 7 years of ART (51). As discussed below, the slightly more rapid decay of QVOA and intact HIV DNA during years 1–7 of ART relative to defective HIV DNA has mechanistic importance and allows mathematical inference of the percentage of cells eliminated by negative selection. Yet, the extremely slow clearance rates of intact HIV DNA and QVOA also suggest that most latently infected cells do not have an overwhelming predisposition toward excess growth or death relative to uninfected CD4+ T cells.

## DEEP LATENCY AND THE PASSENGER HYPOTHESIS

Systemic HIV replication is usually observed within weeks after ART is interrupted (17). This reflects the large number of cells with potentially inducible provirus. However, at the single-cell level, full lytic reactivation is rare and is estimated to occur only once every 14 years (55–57), because most measured provirus is in a state of deep latency. This is most clearly demonstrated by the difficulty in reversing HIV-1 latency *ex vivo*. One of the strongest HIV-1 latency reversing strategies is combined stimulation with PMA and the calcium ionophore ionomycin (I). PMA/I activates similar downstream signaling cascades induced by T cell activation yet bypasses many of the regulatory steps proximal to the T cell receptor (TCR) (58). In doing so, PMA/I permits intense activation of several host transcription factors that stimulate the HIV-1 promoter, including NF-κB and NFAT (59, 60). A crucial observation was that even with maximal stimulation of patient-derived CD4+ T cells, a significant fraction of intact proviruses was refractory to latency reversal with PMA/I (1). Moreover, proviral induction often required many rounds of stimulation (1). While HIV RNA can be detected in latently infected cells during ART (61, 62), recent limiting dilution experiments have estimated that, on average, only 15% of infected cells are induced to express HIV-1 RNA with PMA/I, and that in most single HIV-1 RNA+ cells, the magnitude of induction is modest (<100 RNA copies/cell) (62, 63). *Ex vivo* supraphysiologic stimulation of CD4+ T cells harboring provirus with anti-CD3/CD28 antibodies also leads to release of viral progeny in only 1.5% of cells (64). Single-cell studies reveal substantial heterogeneity among latently infected cells harboring HIV provirus according to epigenetic profiles, transcriptional patterns, activation status, programmed cell death, and CD4+ T cell subset, within and between infected PWH (65–67), which likely explains the wide gradient of HIV reactivation potential across infected cells. Overall, these findings suggest that during sustained ART, chromosomally integrated HIV provirus only sometimes impacts the natural birth and death processes of its host cell. The dynamics of uninfected CD4+ T cells are therefore vital to understanding HIV persistence on ART.

## LIMITED IMMUNOGENICITY OF MOST RESERVOIR CELLS AND THE PASSENGER HYPOTHESIS

Latently infected CD4+ T cells can be targeted for elimination by immune responses, an observation which is being therapeutically leveraged in attempts to eradicate the reservoir (68–70). As discussed below, clear signatures of immune selection are evident during sustained ART (71–74). Yet, the cumulative impact of anti-reservoir immunity is insufficient to induce significant spontaneous elimination of most reservoir cells. HIV-specific CD8+ T cell levels correlate with HIV RNA and DNA levels in PWH on ART but only predict proviral clearance rates during early ART; after a year of ART, there has been no observed relationship between host immune parameters and reservoir clearance rate, which shows limited variability among PWH (75, 76). This suggests that cytolytic T cells recognize but do not frequently eliminate reservoir cells. CD4+ T cells harboring defective HIV DNA transcribe and translate viral genes leading to cell surface expression and recognition by cytotoxic lymphocytes (77). Yet, clones harboring intact HIV DNA appear more resistant to autologous CD8+ T cell elimination (78), despite not harboring more cytotoxic T lymphocyte escape mutations relative to clones with defective HIV DNA (35). Recent single-cell analysis demonstrates that latently infected cells have transcriptional programs compatible with immune evasion (71). Accordingly, as discussed below, our models estimate that immune targeting is a much less common cause of reservoir cell death than mechanisms of CD4+ T cell death which are independent of latent HIV infection (79, 80).

## RESERVOIR CELL PROLIFERATION AND THE PASSENGER HYPOTHESIS

To optimize strategies for a functional cure, it is necessary to understand core mechanisms sustaining the reservoir. Provirus sequencing is an essential tool for this task. Given the high error rate of the HIV reverse transcriptase enzyme and the ~3 billion possible chromosomal integration sites for HIV DNA, if an HIV integration site or viral DNA sequence is only present in one cell in the entire body, then this singleton sequence was probably generated by a unique viral infection of this cell (though the cell could also be a member of a waning proliferative clone as defined below) (81). If the HIV integration site and corresponding HIV provirus sequence are equivalent in at least two cells, then these sequences were generated by the less error-prone cellular DNA polymerase during mitosis. These cells are likely members of a proliferative clone generated by rounds of cellular proliferation. In the literature, it is common to use either integration sites alone or near-full-length HIV provirus sequence as barcode surrogates of uniqueness (16, 32, 33).

The drug sanctuary hypothesis of reservoir persistence posits that ART is imperfect, and that low-level viral replication and infection of new cells occurs in deep lymphatic tissues (82). If this were true, data signals for this phenomenon would include continued observation of evolving singleton sequences despite ART. Drug development focused on better delivery of ART to sanctuary sites would be a critical cure strategy (83). Yet, evidence for a drug sanctuary is lacking. While accrual of several apparent new mutations may be observed during the first 6 months of ART, modeling suggests this is likely a fossil record of cellular infections within longer-lived cells prior to ART (84–86). HIV provirus sequence evolution is typically not observed after the first 6 months of ART (22, 84, 87), including in lymphatic sites (88, 89). While ongoing low-level infection cannot be ruled out given the profound undersampling of HIV provirus sequences, if a drug sanctuary exists, then different models suggest it accounts for a minority of reservoir cell formation during sustained ART (86, 90).

The clonal hypothesis suggests that CD4+ T cells in the reservoir persist due to cellular proliferation. Multiple studies demonstrated HIV proviruses with identical integration sites or sequences, or the best evidence yet—identical paired integration sites and provirus sequences—from blood draws and within infected tissues (22, 91–94). Seminal proof of concept studies showed multiple equivalent chromosomal integration sites in 10 to 60% of observed HIV DNA sequences (16, 32, 33). The remaining observed singleton sequences, in theory, allowed a possible contributory role for the drug sanctuary hypothesis. In this case, a multifactorial etiology for reservoir preservation would be important to consider, as this would warrant that HIV cure strategies target both mechanisms of persistence.

A significant and unavoidable issue for all HIV reservoir sequencing studies is massive undersampling, which is compounded by trafficking of reservoir sequences between blood and lymphatic tissues (95, 96). Even deep sequencing from leukapheresis, in which peripheral blood mononuclear cells (PBMCs) from a volume of blood much larger than a typical blood draw are collected, likely captures less than 1 millionth of total body HIV DNA (86). To address this problem, we pioneered reservoir ecology to infer the structure of the whole-body reservoir (86, 97). By generating theoretical distributions of the entire reservoir, subsampling from these distributions and fitting to observed data, we concluded that at minimum 99% of defective and intact HIV DNA sequences were clonal in origin, and that most (and likely all) observed singletons were members of proliferative clones (97, 98).

A more mechanistic model suggested that as many as 5% of new infected cells could be generated by viral replication without being detected as new sequences in phylogenetic trees (90). Indeed, one limitation of our ecologic approach is that it assumes the unobserved tail of this distribution follows the same slope as the observed portion and that sequences in blood and tissues are well mixed. Extrapolated rank abundance estimates have generated false predictions of species abundance for other biologic systems (99–101). Accordingly, our approach cannot confidently estimate the number of unique sequences in the reservoir but does predict that most of these sequences exist within clones (97, 98).

**Fig 1:**
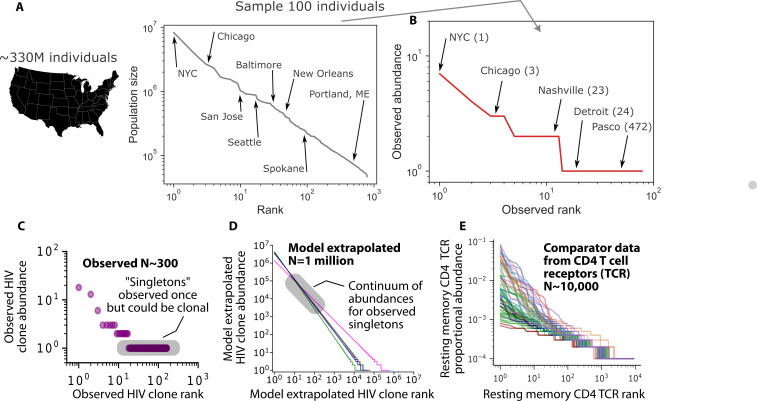
Analogy to city sizes: HIV clone distributions resemble those of carrier CD4+ T cells. (**A**) Rank abundance of USA by city where each city is ranked from largest to smallest by population size. A power law (log-log-linear) relationship is observed. The rank of certain cities of interest is annotated. (**B**) Randomly sampling 100 individuals from the USA shows that large cities are repeatably represented, but even highly ranked cities are often not observed, and medium/low ranked cities are usually missed, but sometimes observed once. Parentheses next to city names denote their true rank in panel (**A**). (**C**) Example observed HIV DNA proviral clone rank abundance from a single PWH on long-term ART—clones are ranked left to right from largest to smallest by abundance. With *N* = 300 sampled sequences, most clones are observed once (shaded gray “singletons”) (86). (D) We extrapolated rank abundances to *N* = 10^6^ cells in the reservoir and projected that observed singletons are likely smaller but nonetheless clonal, existing on a continuum of abundances (shaded). (**E**) Comparator rank abundances of resting memory CD4+ T cells from 37 HIV-neg people and 11 PWH on ART based on TCR sequencing (79). The similar power law structure suggests CD4+ T cell clone distributions drive HIV reservoir distributions via cellular proliferation.

Across numerous time points of sampling during ART and in multiple PWH, the HIV reservoir is predicted to consist of an organized structure consisting of a small number of massive clones and a massive number of small clones (35, 86). This finding has also been observed in lymphatic compartments (88, 102), where viral evolution indicative of ongoing infection also stops concurrent with ART initiation (89). An HIV reservoir clone’s rank in size may be roughly predictive of its actual size, a relationship that is remarkably similar to ranked population size of cities and towns in the USA (Fig. 1A). Nearly all ~335,000,000 US citizens live in one of a small number of large cities or in one of the tens of thousands of small to moderate-sized towns. Akin to typical reservoir studies, if only 100 citizens were sampled, then most towns would not be captured, and the small fraction that were would be observed only once (Fig. 1B). Moreover, most sampled people would misleadingly appear as observed singletons despite the fact that they reside in a heavily populated city or small town. In our simulation of this experiment, New York and Chicago (ranks 1 and 3) are sampled 7 and 3 times out of 100. Despite San Jose and Seattle being ranked 10th and 18th, they are not sampled once in this random subsampling of 100 Americans. Detroit (rank 24) and Pasco, Washington (rank 472) are both sampled once. These analogies demonstrate how massive undersampling inevitably misses large clones while randomly capturing a tiny minority of smaller ones. Still, the precise shape and slope of the tail of these distributions remain unknown due to the under-sampling problem.

Studies of T cells using TCR sequencing suffer from undersampling to a lesser degree as hundreds of thousands of cells can be analyzed. In people with and without HIV, TCR rank abundance curves demonstrate a similar rank abundance relationship to our ecologic projections of the HIV reservoir (Fig. 1C through E) (79, 92). This similarity broadly supports the concept that clonal dynamics of most HIV reservoir cells reflect those of uninfected CD4+ T cells. An elegant study that identified the T cell receptor and the HIV provirus integration site from several unique infected clones demonstrated that CD4+ T cell clones can harbor different proportions of infected cells within the clone, suggesting that HIV infection of a member of a clone may occur early or late during clonal expansion or contraction (103). Another study demonstrated that infected CD4+ T cell clones are somewhat more stable in size over 2–3 years relative to uninfected clones (97) (though many infected clones notably wax and wane [104, 105]), perhaps because all infected cell clones are seeded prior to ART, whereas uninfected CD4+ T cell TCR clones continue to emerge after ART.

## PROLIFERATIVE CLONES AS A SOURCE OF REBOUND VIREMIA

The importance of cellular proliferation in maintaining inducible reservoir cells is supported by the fact that rebounding HIV has been observed from previously detected reservoir clones (106–109). This is remarkable given that >99.9% of clones are undetected due to undersampling in typical HIV reservoir studies (86). Nonsuppressible residual viremia also appears to emerge from proviral clones containing 5´-leader defects which render the amplified viral RNA non-infectious (110). Unsurprisingly, most detectable rebounding virus is not from previously identified sequences (111–114). Intriguingly, there is an inverse relationship between clone size and viral outgrowth in culture (114). This may be partially explained by the fact that several of the largest defective single HIV DNA clones are greater in size than the entire population of genetically intact HIV DNA proviruses during persistent ART (35, 38, 61, 79, 86, 115).

Proliferation and activation of latently infected cells are often coupled with HIV reactivation within specific CD4+ T cell subsets (65). However, only a small proportion of infected cells within reservoir clones *in vivo* express unspliced HIV RNA following proliferation (116). Reservoir cell proliferation is estimated to occur several hundred times more frequently than a full HIV reactivation event (55). This is key evidence that normal CD4+ T cell physiology more commonly impacts cellular dynamics than integrated HIV provirus.

## PROGRAMMED CELL DEATH AND THE PASSENGER HYPOTHESIS

An important and often overlooked component of cellular proliferation as the sustaining generative force of the reservoir is that the cellular proliferation rate must be matched with an equivalent death rate to account for lack of significant expansion in reservoir volume over years in virtually all individuals who have been followed on chronic ART (55). Elegant single-cell analyses have shown pro-survival signals in latently infected CD4+ T cells, including inhibition of death receptor, necroptosis, and anti-proliferative signaling (117, 118). It is possible that latent infection increases the lifespan of infected CD4+ T cells, but this would need to be matched by a decrease in proliferation rate to explain observed stable reservoir volume. During very late ART, the intact reservoir may grow extremely slowly, doubling in size every 23 years (51). As an upper bound on normal reservoir growth, a recent case report noted slow reservoir expansion by three- to sixfold over a decade in a PWH with no obvious clinical manifestations (119).

The HIV reservoir typically has no features of a lymphoproliferative disorder in which potentially harmful hematopoietic cell levels increase at clinically meaningful levels over time. While immunosuppression and poor control of emergent tumors is common during uncontrolled HIV infection and persists even after years of ART, there have been only a few documented T cell lymphomas derived directly from HIV-infected cells. These tumors were notable for HIV DNA integration into signal transducer and activator of transcription 3 and lymphocyte-specific protein tyrosine kinase genes, which was likely the first step in a multi-hit carcinogenic process (120). In most cases, the frequent integration of proviruses into tumor suppression genes is not pre-malignant (32, 33). Clonal hematopoiesis, a clinical syndrome which refers to an excess of hematopoietic stem cells and precursors, is more common in PWH and is associated with lower survival rates and higher degrees of CD4+ T cell depletion (121, 122). Yet, clonal hematopoiesis does not arise from infected cells. Overall, the long-term stability of the reservoir suggests that latently infected cells may die via the same programmed cell death mechanisms and at similar rates to uninfected, normally functioning CD4+ T cells (123, 124).

## INDIVIDUAL CD4+ T CELL CLONES AS HIV RESERVOIR BUILDING BLOCKS

The long-term stability of proviral defective and intact HIV DNA levels on ART and the reservoir’s organized clonal structure obscures the fact that the reservoir is highly dynamic. The reservoir is composed of tens of thousands of individual proliferative clones which frequently change in size (86, 104, 105, 125–127). CD4+ T cells undergo homeostatic proliferation, which is a generalized mechanism to maintain immune memory and readiness and is balanced with cell death (128, 129). When a memory CD4+ T cell recognizes its cognate antigen being presented by a professional antigen-presenting cell, it undergoes a rapid burst of clonal proliferation as part of a recall response (103). T cell division occurs every few hours in this context (130). Given that certain large HIV-infected CD4 clones likely consist of more than 10 million cells (86, 97), it can be inferred that over 20 rounds of consecutive cell divisions occurred to generate these clones. Following proliferative bursts, CD4+ T cell clones slowly contract over months prior to re-establishing a steady state (123). The frequency with which reservoir clones undergo antigen-driven proliferation depends on the antigen reactivity of the cell (63), and the frequency with which that infected cell is exposed to its cognate antigen. While antigen-responsive clones to HIV, cytomegalovirus (CMV), Epstein-Barr virus (EBV), and influenza antigens have been identified in the reservoir, these represent a minority of observed clonal sequences (103, 131, 132). The proportion and type of antigen-specific reservoir cells vary significantly across PWH (103, 131, 132). Engagement of CMV and HIV cognate antigen is associated with increased HIV transcription, albeit only in a small proportion of CD4+ T cells which are activated and proliferate with effector molecule expression (63). The rarity of antigen-specific cells and the low inducibility of HIV provirus in most of these cells again supports the mechanisms sustaining uninfected CD4+ T cell populations as critical to reservoir sustainment.

## CD4+ T CELL SUBSET DIFFERENTIATION AND THE PASSENGER HYPOTHESIS

**Fig 2:**
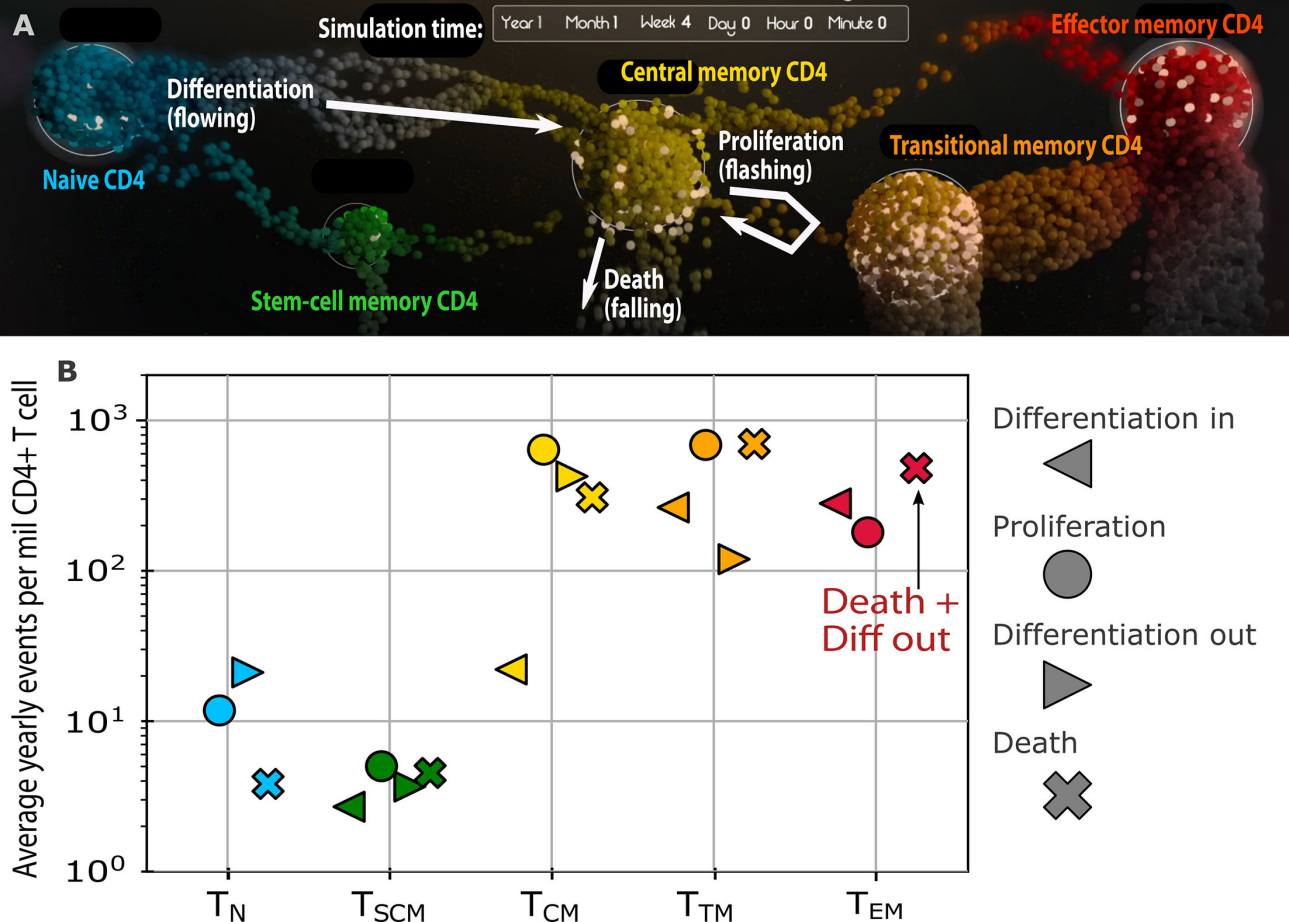
Estimating the magnitude of forces governing HIV reservoir clones (proliferation, differentiation, and death). (A) Mathematical model inferred from data on HIV DNA levels in PWH on long-term ART who ingested deuterium for cell labeling studies of five CD4+ T cell subsets. (**B**) Magnitude of the four generative and elimination forces estimated for each CD4+ T cell subset (80, 133). (Both figure panels republished from reference 80.)

Multiple CD4+ T cell subsets compose the HIV reservoir (5, 42, 133), which is relevant because infected naïve (T_N_) and stem cell-like memory (T_SCM_) CD4+ T cells turn over (i.e., proliferate and die in balance) less frequently than central memory (T_CM_), transitional memory, and effector memory (T_EM_) CD4+ T cells. T_N_ and T_SCM_ also represent a smaller and less clonal portion of the reservoir and may be less likely to reactivate HIV (125, 134). Reservoirs with a greater proportion of T_N_ have greater sequence diversity and fewer large clones (135). Our mathematical modeling of longitudinal data demonstrated that over several years of ART, HIV DNA levels remain stable in all subsets other than terminally differentiated CD4+ T cells in which levels slowly decline (80). Pharmacologically “pushing” memory cells into terminally differentiated subsets could therefore accelerate reservoir elimination and enhance latency reversal attempts (136). We also estimated that the movement of CD4+ T cells between memory subsets is nearly as frequent as proliferation, suggesting that normal CD4+ immune recall responses are a key component of reservoir dynamics (Fig. 2) (80). Modeling also predicts that an effective anti-proliferative therapy would eliminate the most frequently proliferating cells, leading to eventual predominance of T_N_ and T_SCM_ CD4+ T cells in the reservoir, suggesting that a shift in reservoir subset composition is a relevant mechanistic clinical trial endpoint (55). Of note, this is the opposite trend of what very slowly occurs during prolonged, sustained ART (134). Deciphering the role of reservoir subset dynamics is complicated by the fact that CD4+ T cells within each subset demonstrate highly variable degrees of activation, proliferation, programmed cell death, and HIV RNA transcriptional signaling (65).

## THE ROLE OF CELLULAR PROLIFERATION IN FORMING RESERVOIR CELLS DURING EARLY UNTREATED HIV

Most studies of the HIV reservoir are performed after at least 6 months of sustained ART, primarily because the reservoir can be sampled without contamination from sequences in short-lived, actively infected cells (85, 86). Because actively infected cells each contain a unique true singleton sequence, the presence of high numbers of actively infected cells prior to ART obscures interpretation of clonal HIV provirus sequence data. Human and non-human primate studies demonstrate that the reservoir is formed within 4 days of infection and that reservoir size inversely correlates with earlier initiation of ART during early infection (44). It is therefore important to consider mechanisms of reservoir cell creation and elimination during primary infection prior to ART.

Primary infection has more complex CD4+ T cell dynamics than chronic ART. Peak viremia at approximately day 7–10 of infection is associated with substantial CD4+ depletion, particularly in gut tissues (137). Blood CD4+ T cell levels then undergo rapid proliferative recovery over the next several weeks. Clonal HIV DNA sequences are observed within 2–3 months of untreated infection despite a concurrent sea of true singleton sequences from actively infected cells (138). A mathematical model showed that to achieve observably large clones despite the overwhelming amount of diverse sequences arising from viral replication, massive proliferation of infected CD4+ T cells is required to occur between weeks 1 and 4 of infection (with highly variable rates of proliferation across clones) (139). The proportion of detected clonal sequences would predictably increase with deeper sequence depth. CD4+ T cell recovery inclusive of cells with and without HIV provirus during untreated primary infection likely represents the first stage of the passenger hypothesis.

## THE ROLE OF CELLULAR PROLIFERATION IN FORMING RESERVOIR CELLS DURING CHRONIC UNTREATED HIV AND EARLY ART

It is similarly challenging to assess reservoir cell dynamics during chronic untreated HIV infection once viral loads have stabilized in plasma, due to the continued presence of a sea of true singleton HIV DNA sequences within recently infected cells. Several studies employed a clever design comparing post-ART HIV DNA sequences with sequential pre-ART plasma RNA sequences to infer the timing of reservoir seeding (87, 140–142). While these studies demonstrated reservoir sequences from all pre-ART time points, a preponderance of reservoir HIV DNA was from the 2 years before ART initiation. Mathematical modeling of these data showed that this distribution could emerge only by allowing older archived reservoir sequences to be cleared and replaced with current sequences. The estimated rate of individual sequence clearance pre-ART was significantly faster (~12 month half-life) than the clearance rate of the reservoir during ART (~44 month half-life) (140). Continual updating of predominant proviral sequences is necessary to explain this discrepancy and is estimated to occur at a rate 15 times higher during untreated HIV relative to sustained ART, perhaps due to more active immune clearance of infected cells as well as more frequent CD4+ T cell turnover pre-ART (143).

The initial event to achieve strain replacement must be new infection of a CD4+ T cell that reverts to latency. To then become a clinically detectable reservoir sequence, subsequent massive clonal proliferation is also a likely pre-requisite (Fig. 1C and D). This suggests that CD4+ T cell proliferation is the major source of reservoir cell generation during ART and likely contributes during early and chronic untreated HIV, though the proportion of reservoir cells generated by proliferation versus infection pre-ART is unknown. There may be a role for anti-proliferative therapies concurrent with or prior to ART initiation.

**Fig 3:**
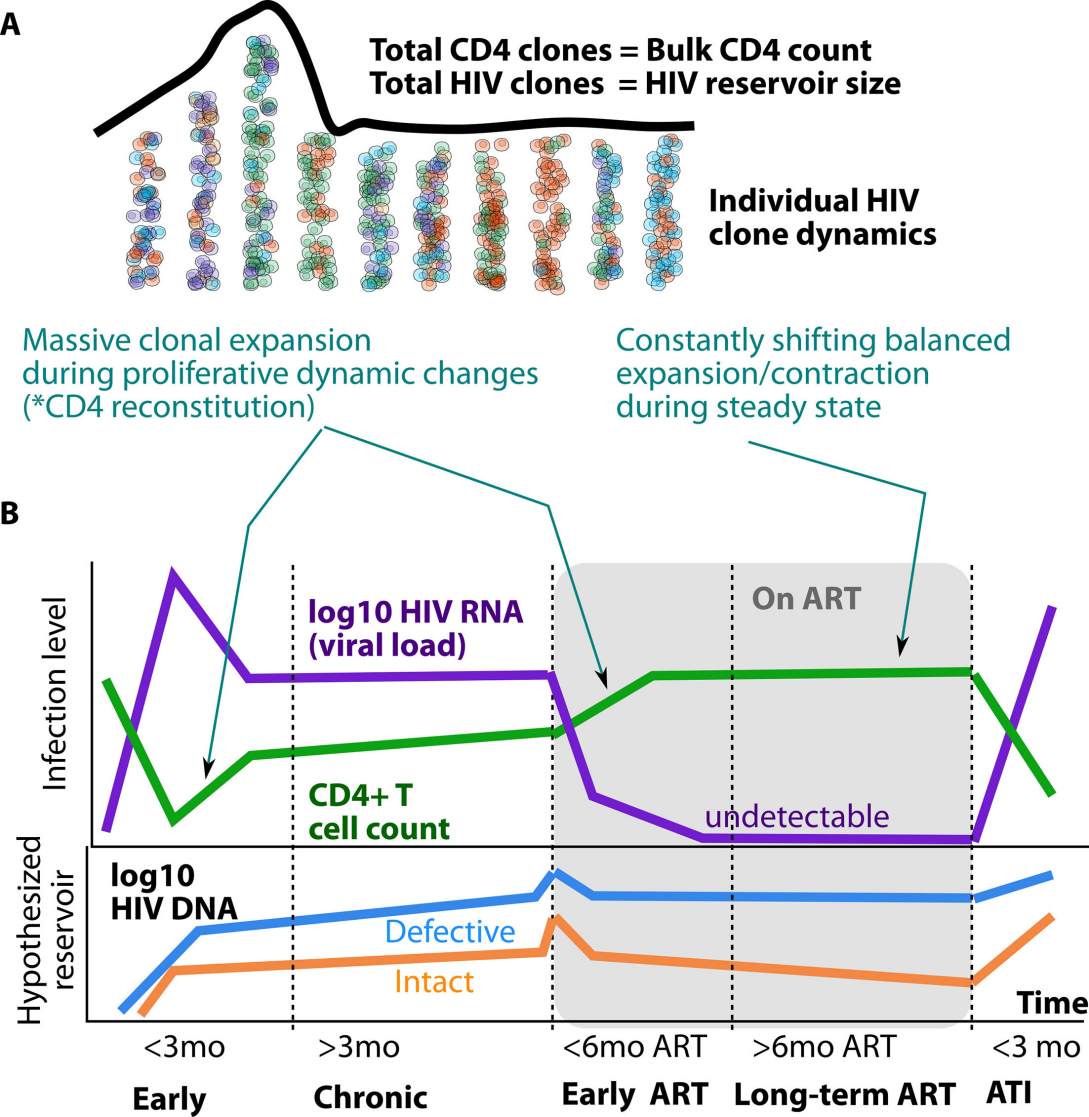
Overall model of HIV reservoir proliferation clone dynamics according to stage of infection. During CD4+ T cell recovery after peak viremia, models suggest that uneven clonal proliferation occurs and accounts for most reservoir cell generation, though many cells continue to be seeded by direct viral infection. During chronic untreated HIV, reservoir sequence turnover is higher than during ART, which may be explained by continual evolution of HIV sequences and ongoing expansion and contraction of reservoir clones. Sustained ART generates a more clonal reservoir as the dominant mechanism driving reservoir cell formation is proliferation. Latent cell proliferation is balanced by death to account for the quasi-stable levels of defective and intact HIV DNA over years of ART. The higher clearance rate of intact sequences is likely due to low-grade negative selection, which may dissipate with time on ART. Rebound viremia following ATI is likely to arise from observed or unobserved clonal sequences of inducible provirus.

Overall, we propose a model of reservoir cell formation which varies according to stage of HIV infection (early untreated, chronic untreated, early ART, and late ART). Differences between these stages of infection can be attributed to differing CD4+ T cell birth and death dynamics (Fig. 3) but consistently relate to HIV being a passenger within CD4+ T cell proliferative clones.

## DEVIATION FROM THE PASSENGER HYPOTHESES: SLIGHTLY INCREASED DEATH RATE OF RESERVOIR CELLS WITH GENETICALLY INTACT PROVIRUS

Despite the critical role played by proliferation, programmed cell death, and subset differentiation in sustaining reservoir volume during chronic ART, there is evidence from elegant single-cell experiments that cells harboring intact HIV DNA have different transcriptional and phenotypic patterns from cells harboring defective HIV DNA and from uninfected CD4+ T cells, though active HIV transcription is necessary to detect these differences (71, 117, 144). Important differential impacts of intact HIV proviruses on host cells can also be observed in reservoir cell dynamics. In multiple cohorts, clearance of intact HIV DNA was slightly more rapid than that of defective HIV DNA during the first 5–10 years of ART (4, 51, 52, 54, 127, 145). In addition, HIV provirus sequences are more clonal overall than TCR sequences from uninfected cells in the same individual (79). Intact HIV DNA clonality also increases with time on ART while TCR clonality remains stable, implying specialized dynamics of reservoir cells (32, 33, 61, 79).

The passenger hypothesis is necessary but insufficient to explain these trends. Using mathematical models applied to both intact HIV DNA and CD4+ T cell clonality data, we estimate that for every 34 latently infected cells harboring intact HIV DNA that die “naturally” between years 2 and 10 post-ART initiation, one dies as a result of harboring intact HIV (79). The stabilization of genomically intact HIV DNA after ~7 years of ART suggests this excess death may diminish with time due to selection (51).

## DEVIATION FROM THE PASSENGER HYPOTHESES: NEGATIVE SELECTION OF PROVIRAL SEQUENCES BASED ON VIRAL INTACTNESS, INTEGRATION SITE, AND INFECTED CELL PHENOTYPE

Two intertwined negative selection mechanisms explain the slightly more rapid death rate of cells containing intact HIV DNA. First, viral reactivation resulting in cytolytic death may explain periodic low-level viral RNA detection in plasma, which diminishes slowly over at least the first 7 years of ART (53, 146, 147). HIV mRNA, gag, and p24 expression can be detected in rare circulating transitional and effector memory CD4+ T cells (67, 148). HIV RNA expression is detectable but low and equivalent between clonal reservoir cells containing defective versus intact HIV DNA. However, most defective viruses have very large deletions and multiple mutations preventing intact HIV protein expression (1). While intact or nearly intact provirus gene expression is likely to be more cytotoxic (62, 149), T cell activation does not predictably reactivate HIV DNA, suggesting that this mechanism of negative selection may be limited to a small fraction of reservoir cells (63), a finding that helps explain why intact HIV DNA declines very slowly during sustained ART (150, 151).

Second, more frequent HIV gene expression and cell surface protein expression, as well as accumulation of intracellular viral replication intermediates, periodically allow latently infected cells to be targeted by host adaptive and innate immune responses (77). The bulk of immune elimination of reservoir cells during chronic ART may be concentrated during early ART, given that intact HIV DNA levels stabilize after 7 years of ART. The lack of CD8+ T cell escape mutations in the reservoir (152), the lack of accumulation of CD8+ T cell escaped epitopes within HIV DNA during ART (35), and the absence of effective CD8+ T cell responses against reactivating HIV (153) (despite the ability of autologous antibodies to prevent rebound) (154) all suggest that a major limitation preventing immune removal of a greater number of infected cells is lack of sufficient HIV expression due to deep latency. Latently infected cells also express cell surface molecules which may further contribute to evasion of lysis by host CD8+ T cells and natural killer cells (71).

Elegant single-cell analyses which incorporate viral sequence, chromosomal integration site, and cell transcriptional profile demonstrate the extent and phenotype of negative selection on the reservoir. During sustained ART, latently infected cells with intact HIV DNA integrated into highly transcribed genic euchromatic regions are gradually pruned, while intact proviruses within transcriptionally silent regions persist, indicating gradual selection (34, 73). Over many years of ART, viruses are preferentially integrated into repressive heterochromatic locations, favoring an overall shift toward deep latency (34, 72). Latency reversal agents accelerate this process by favoring persistence of sequences integrated in centromeric satellite DNA and ZNF genes (155). The reservoir assessed with multi-probe PCR and QVOA steadily becomes more clonal during ART (35, 73, 79, 127), suggesting that the remaining latently infected cells are associated with less lytic viral replication and immune targeting following cellular activation and proliferation (155). Elite controllers who are able to naturally suppress viremia without ART demonstrate a similar pattern, with lower levels of intact HIV DNA that is concentrated within clonal populations of CD4+ T cells in heterochromatin repressive chromosomal regions distant from transcriptional start sites, suggesting strong immune selection against transcriptionally active cells (74, 156, 157).

Aside from the integrated virus, the phenotype of the infected cell may also drive negative selection. The “original sin” hypothesis posits that the specific cells likely to be infected by replication-competent HIV infected pre-ART are more prone to viral integration into euchromatic regions relative to HIV-exposed uninfected CD4+ T cells which remain uninfected. These cells may be a specialized subclass of CD4+ T cells notable for CCR5 expression, greater subset differentiation, and higher rates of proliferation and death relative to uninfected and less activated CD4+ T cells (137, 158, 159). If the natural death rate of this class of cells slightly exceeds their proliferation rate independent of harboring HIV, then the passenger hypothesis could explain differential decay of intact viruses. Upon achieving latency in an *ex vivo* system, cells with intact HIV DNA proliferate less avidly than uninfected cells and cells containing defective HIV DNA, which might reflect a bias in the phenotype of cells infected with intact virus (160). Reservoir clones also appear to be less dynamic than uninfected CD4+ T cell clones, which might imply a specialized cell population, but may also reflect that formation of new reservoir clones is halted with sustained ART while new clones of uninfected CD4+ T cells continue to emerge (97).

## DEVIATION FROM THE PASSENGER HYPOTHESES: POSITIVE SELECTION OF PROVIRAL SEQUENCES BASED ON VIRAL INTACTNESS, INTEGRATION SITE, AND INFECTED CELL PHENOTYPE

Proviral integration sites also may contribute somewhat to positive selection given a slight observed trend during ART favoring proliferation of proviral sequences integrated into specific oncogenes, albeit in a small fraction of detected clones (32, 33, 161). A shift toward pro-survival cell surface molecule expression with extended time on ART has also been observed (71). Among persistent defective proviruses, the presence or absence of certain intact viral genes may also favor the relative predominance of a given sequence (162). These trends suggest that viral intactness and integration site may allow certain proliferative clones to outcompete others, even in the absence of negative immune selection.

## HIV CURE INTERVENTIONS

The primary importance of the passenger hypothesis is either as a barrier or facilitator of HIV cure. The goal of the HIV cure field is to allow PWH to stop ART without the risk of reactivating HIV. HIV eradication is defined as no detectable HIV DNA or RNA in the body. HIV functional cure is defined as no sustained HIV RNA viremia off of ART, even if residual intact or defective HIV DNA is detected in blood or tissue samples. Functional cure may occur if the reservoir is reduced to a sufficient degree to significantly lower the probability of reactivation (55–57). The precise threshold of reservoir volume that would confer durable remission has been estimated using modeling (56, 57), but validation of these estimates is beyond current capabilities due to the limited efficacy of current curative therapies. Even PWH with very small reservoirs exhibit viral rebound (163), and data in rhesus macaques suggest that a 100-fold decrease in simian immunodeficiency virus (SIV) DNA leads to only a twofold decrease in SIV reactivation rates after ART withdrawal (164).

Post-treatment control is defined as innate or adaptive immune-mediated clearance of all HIV reactivations despite persistence of a detectable and inducible reservoir and may lead to functional cure (165, 166). Post-treatment control has been attained in a select few preclinical studies and is observed naturally on occasion (69, 167, 168). The goal of a functional HIV cure is for PWH to live without taking ART with only periodic monitoring for recrudescent viremia and CD4+ T cell stability.

Cure studies should always include longitudinal assessment of reservoir volume using assays for total and intact HIV DNA, with possible use of QVOA, while ART dosing continues (49). ATI of ART is also often built into studies and is necessary to diagnose functional cure. Even if HIV reactivation occurs following ATI, delayed time to HIV RNA detection, slower HIV RNA rebound kinetics, or lower viral load peak or setpoint (biomarkers that might correlate with a smaller or pharmacologically altered reservoir) may indicate partial therapeutic effect of an intervention (7, 169, 170). For these purposes, the testing of HIV cure strategies in non-human primates may provide useful information, as ART treatment interruptions can be performed in preclinical models.

## THERAPEUTIC CHALLENGES POSED BY THE PASSENGER HYPOTHESIS

There have been multiple attempts to use latency reactivation agents (LRAs) to induce viral gene expression of infected cells or to enhance their targeting by endogenous or therapeutically enhanced immune responses (171). LRAs have induced transient detectable viremia with different degrees of potency, but with no evidence of reservoir reduction (151, 172). Recently, panobinostat appeared to delete activated proviruses and enrich for deeply latent proviruses impacting clonal structure, though the proportion of cells sensitive to this LRA was too low to reduce reservoir volume (155). The most simple explanation is that too few cells are reactivated following each dose due to deep HIV latency, in keeping with the passenger hypothesis. Another possibility is that reactivating cells are killed but also proliferate in response to being stimulated, leading to no net change in reservoir size. A related issue is that sufficiently potent stimulation of reservoir cells to achieve reactivation might lead to untenable systemic toxicity. Selection of deeply latent proviruses in gene desert chromosomal sites signifies that populations of naturally reactivating active cells are impacted by immune pressure but may contribute to the challenge of reactivating viruses sufficiently to eliminate infected cells, particularly in PWH on prolonged ART (173).

Immunotherapies are under intense study for HIV cure (173). Augmentation of endogenous HIV immunity through vaccination, neutralizing antibody infusion promoting cytolytic T cell responses, or exogenous cellular immunotherapies such as CAR T cells show promise and could accelerate pre-existing negative selection of reservoir cells (174–176). Each approach could in theory be augmented by antecedent potent latency reactivation. Yet, if the central feature of the passenger hypothesis is true, that most latently infected cells exhibit dynamics similar to those of uninfected CD4+ T cells, then this implies most reservoir cells exist in a deep state of latency with limited immunogenicity. One promising method to bypass this challenge may be to dose immunotherapies prior to or during ART initiation to promote robust memory immune responses that persist when HIV antigen is later present in limited abundance during sustained ART (175, 176).

Another strategy termed “block and lock” intends to push HIV into even deeper latency (177, 178). This is an attractive approach to achieve a functional cure, but it should be noted that the passenger hypothesis suggests it will not reduce the size of the reservoir as these cells will continue to proliferate and die at equal rates. Block and lock would need to be sustained indefinitely in PWH, making it similar in practice to long-acting ART. Gene therapy approaches intend to target chromosomally integrated HIV DNA with various cutting enzymes (179). This approach should be less hindered by underlying CD4+ T cell dynamics, provided viral delivery vectors do not activate proliferative pathways. Yet, delivery of such approaches to every applicable cell and ascertaining no off-target genomic cutting are technologically challenging (180).

## ANTI-PROLIFERATIVE THERAPY FOR HIV CURE

If cellular proliferation is the fundamental mechanism sustaining HIV reservoir cells, then it is an attractive target for curative interventions. Mathematical models suggest a bathtub analogy in which water levels are quasi-stable if there is a constant inflow of water through a slightly open faucet (cellular proliferation) with equal outflow through a narrow drain (cell death). Turning off the faucet is akin to anti-proliferative therapy and would allow the bath water to deplete based on the outflow rate through the drain (the natural death rate of CD4+ T cells) (55). The water level would decrease proportionally to the level of bath water in keeping with exponential decay.

**Fig 4:**
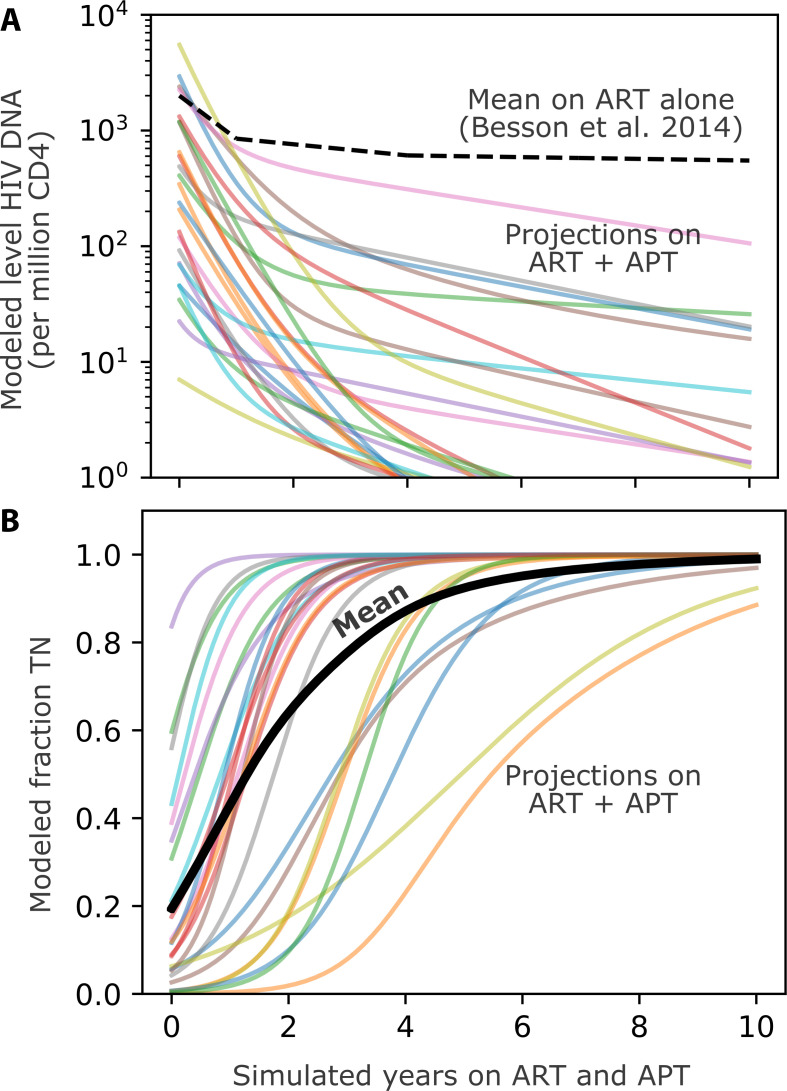
Projections of ART+APT based on models of participants in the HOPE study. Using a mathematical model informed by cell proliferation rates and HIV DNA in five CD4+ T cell subsets (T_N_, T_SCM_, T_CM_, T_TD_, T_EM_) from 37 individuals with varying clinical histories of ART, we projected HIV DNA levels during 10 years of anti-proliferative therapy (APT, proliferation rates reduced twofold) in addition to ART (80, 133). (A) Projections of HIV DNA levels summed across all subsets: each colored line denotes a projection based on individuals with experimentally derived levels of HIV DNA as well as proliferation rate of cells in each CD4+ T cell subset. The dashed black line provides a comparison for typical HIV DNA kinetics on long-term ART. (**B**) The fraction of naïve CD4+ T cells (T_N_) rises during these projections because these cells proliferate less frequently, and thus are reduced less by APT.

Mathematical models which assume variable initial reservoir levels and variable fractions of HIV DNA in each subset, and incorporate experimentally derived CD4+ T cell turnover rates of each subset (derived from heavy water labeling studies in PWH), suggest that anti-proliferative therapy could lead to a 2–3 log depletion of HIV DNA over the course of 1 year of continuous therapy (Fig. 4A), provided the therapy decreases proliferation by 50% (55). These models also predict biphasic clearance, due to a slower clearance rate once less frequently proliferating subsets such as T_N_ and T_SCM_ predominate in the reservoir, as well as accumulation of HIV DNA in these subsets (Fig. 4B).

Several small studies highlight the promise of this approach. The lymphocyte anti-proliferative agent mycophenolate mofetil (MMF) is the prodrug of mycophenolic acid, a non-competitive inhibitor of inosine-5´-monophosphate dehydrogenase, required for synthesis of the free nucleotide guanosine-5´-monophosphate, which leads to inhibition of DNA replication in lymphocytes. MMF is approved as a steroid-sparing agent for rheumatologic conditions, to prevent graft rejection following solid organ transplantation, and to prevent graft versus host disease following hematopoietic stem cell transplantation (HSCT) (181). In one small trial, 6 months of MMF treatment (500 mg twice daily) was associated with 1–2 log reductions in reservoir levels (by QVOA) in three out of six PWH (182). In another, 120 days of MMF (250 mg twice daily) was started in nine individuals after a year of ART followed by an ATI and continuation of MMF. Six other individuals maintained the same ART and ATI schedule but did not receive MMF. The plasma from six of nine participants receiving MMF inhibited cellular proliferation. Five of these six individuals had an HIV RNA level <200 copies/mL 6 months after ATI versus only one of the other nine participants. This suggests that inhibition of T cell proliferation might have been associated with reservoir reduction leading to delay in time to rebound (183).

More recently, our group studied 1 year of MMF treatment in four PWH. MMF dose was 500 mg twice daily but was increased to 750 mg twice daily in one individual whose plasma did not inhibit T cell proliferation. We observed no reduction in total or intact HIV DNA, and no shift in reservoir subset composition in any participant (42). A possible explanation for the lack of reservoir reduction was underdosing. We observed a potent anti-proliferative effect of participants’ serum against their own PBMC 30 minutes after dosing levels, but this effect was negligible at drug trough, suggesting the need for an agent with a longer half-life (42). It is possible that a reduction in proliferation was compensated for by an interleukin-7 (IL-7)-mediated negative feedback loop leading to longer-lived reservoir cells (184–186), though IL-7 levels were stable in trial participants at drug trough time points.

Sirolimus (also known as rapamycin) is an inhibitor of mammalian target of rapamycin which blocks cell cycle progression while also limiting T cell activation. Sirolimus has been associated with decreased total HIV DNA levels following renal transplantation (187). Sirolimus demonstrated broad transcriptional changes associated with decreased T cell activation and proliferation but did not lower SIV DNA levels in rhesus macaques (188). A recent single-arm trial tested 20 weeks of sirolimus in ART-suppressed individuals. Among eight individuals with detectable intact HIV DNA at study onset, a median 0.4 log reduction was observed after 20 weeks along with a decreased expression of cell turnover markers, suggesting a partial anti-proliferative effect (189). In another study, methotrexate, an antifolate compound that blocks T cell activation and DNA synthesis and is approved as an immune suppressant and chemotherapeutic agent, lowered total CD4+ T cell counts and lowered cell cycling (190). It remains to be seen whether the slight overall observed reduction in reservoir levels pertains to suboptimal drug effect, subtherapeutic dosing, compensatory pro-survival cell signaling, or overall limitations of this approach.

## A DRUG DEVELOPMENT PIPELINE FOR ANTI-PROLIFERATIVE THERAPIES

Each of the aforementioned studies was small and informed by limited preclinical data. We contend that proper evaluation of lymphocyte anti-proliferative agents requires a more structured approach akin to that used for small molecule drug development for ART and LRAs. We recently initiated the first step of this process by screening a large library of licensed cancer compounds for inhibition of T cell proliferation (191).

Of those tested, dasatinib emerged as a candidate with potent inhibition of both homeostatic and antigen-driven proliferation at clinically achievable drug concentrations. Dasatinib is a tyrosine kinase inhibitor (TKI) used for Philadelphia chromosome-positive chronic myelogenous leukemia (CML) (192). Dasatinib’s strong inhibitory effects on T cell proliferation were previously recognized (193). TKIs and dasatinib in particular have been shown to have anti-HIV activity during acute infection (194), protected mice in HIV challenge studies, and may prevent latent HIV reactivation. Dasatinib lowers homeostatic proliferation-related cytokines IL-7 and IL-15 *in* vivo (195), and may block HIV replication through the SAMHD1 (SAM domain and HD domain-containing protein 1) pathway by inhibiting the synthesis of deoxynucleotide triphosphates, essential building blocks for HIV reverse transcription (196, 197). Dasatinib is considered mildly immunosuppressive but is not commonly associated with opportunistic infections (198).

Recently, two PWH on ART in Spain were treated with dasatinib for CML. These individuals had 2 and 3 log reductions in total HIV DNA over 1 year (199), as well as biphasic decay as predicted by the model above (Fig. 4A) (55). These reductions were maintained for several months after dasatinib was stopped. To our knowledge, no cure intervention other than stem cell transplantation has induced this level of reservoir depletion. Other PWH receiving dasatinib demonstrated fivefold reductions in total HIV DNA, as well as reductions in levels of the homeostatic cytokine IL-7 (195). Hence, there is justification for controlled human clinical trials of dasatinib. Several pilot trials are being conducted in Europe (NCT05780073 and NCT05527418) and the USA (NIH ACTG trial A5413, “Dasatinib for HIV-1 Reservoir Reduction”). Each has important exploratory components, including analysis of HIV reservoir CD4+ T cell subset composition and evaluation of other possible mechanisms of action, including natural killer (NK) cell activation.

The development pathway for anti-proliferative agents should be expanded. We advocate for highly monitored studies of PWH on ART, with dosing and study endpoints informed by mathematical models. If early human trials demonstrate partial efficacy, then combinatorial approaches with other reservoir reduction strategies should be considered.

Drawbacks of lymphocyte anti-proliferative strategies are important to mention. All trials of anti-proliferative agents must proceed with caution based on the potentially immunosuppressive nature of these drugs. Agents which impact reservoir cells may also impact the size and functionality of the larger uninfected CD4+ T cell population. It is notable that CD4+ T cell lymphopenia is not a commonly reported side effect of MMF or dasatinib, including in PWH (187, 195, 200). Whether this reflects a lack of sufficient anti-proliferative activity to affect total T cell levels, the presence of a compensatory mechanism, or trafficking of T cells from tissue to blood is unknown.

There are several reasons for potential therapeutic failure. *Ex vivo* evidence shows that the largest observed clones may be refractory to HIV reactivation and further proliferation, such that the smallest clones are most relevant for therapeutic targeting (114, 160). Moreover, CD4+ T cells with intact virus may proliferate less avidly upon TCR stimulation, meaning that anti-proliferative agents may preferentially affect non-reservoir cells (160). Yet, the fact that intact sequences are detected at all suggests that they exist within a large clone. It is possible that some of the largest observed clones from human samples are in a programmed contraction phase and are refractory to further proliferation. On the other hand, CMV stimulation appears to induce proliferation in already large antigen-specific clones (63). It is possible that small clones which are about to undergo large proliferative bursts are key targets of sustained anti-proliferative strategies but largely remain undetected due to profound undersampling (86).

Careful endpoint selection for anti-proliferative therapy trials is important. Recent evidence from coronavirus disease 2019 trials demonstrates that viral clearance slope derived from multiple sequential measurements is more sensitive to detect a drug effect than absolute reduction in viral load (201). A similar statistical approach could be applied to cure studies to maximize statistical power. Additionally, we showed that minor issues of specificity in multi-probe intact proviral DNA assays (IPDAs) can be magnified if therapies preferentially clear intact proviruses such that observed intact levels might be mostly false positives if a therapy is effective (47).

## TARGETING LARGE VIRUS-SPECIFIC CLONES

Indirect approaches that relate to reservoir clonal proliferation are being considered. Co-infection with CMV and EBV has been associated with higher HIV reservoirs in pediatric cohorts (202). Large reservoir clones have repeatedly been found to have TCR that are CMV-specific (79, 103, 131). Thus, a trial using the antiviral letermovir to limit the presence of CMV antigen is ongoing (NCT04840199). As a proof of principle, this trial is informative and has potential broad impact given high CMV seroprevalence in many HIV-infected populations (203). Yet, it does not address the many clones that are specific for EBV, influenza, HIV, gut bacterial peptides, or other undiscovered antigens. Moreover, the largest CMV clones may have experienced their burst phase early during life and may not be sensitive to anti-proliferatives (204). Finally, letermovir has no role in CMV-seronegative individuals.

Settings in which this approach could hold promise would be patient populations with small reservoirs, subsets of HIV-1 elite controllers, or PWH who have been on ART for very long amounts of time. In each of these settings, detectable reservoirs in blood may consist of only a few highly expanded proviral clones (72, 157). Nonsuppressible HIV-1 viremia (NSV) is defined as persistent low-level viremia on ART without evidence of drug resistance. The infected cells responsible for NSV are often single clones that are highly expanded in circulation (110, 205), highlighting an additional clinical setting in which clone-specific targeting could hold utility.

## PRO-APOPTOTIC THERAPY FOR HIV CURE

A higher death rate of reservoir cells could also deplete the reservoir. Revisiting the bathtub analogy, this approach equates to widening the drain to more rapidly lower the water level. This hypothesis is being assessed in an ongoing clinical trial of venetoclax, a BCL-2 inhibitor used for chronic lymphocytic leukemia which triggers apoptosis (206). If this trial demonstrates meaningful reservoir reduction, then it is a plausible hypothesis that anti-proliferative therapies and pro-apoptotic therapies may synergistically increase the clearance rate of the HIV reservoir. Concerns relate to drug toxicity as well as compensatory CD4+ T cell proliferation that may repopulate the reservoir. Ideally, CD4+ T cell levels would be maintained by thymic emigration.

## PRO-DIFFERENTIATION THERAPY FOR HIV CURE

Since most reservoir clearance appears to occur in terminally differentiated effector memory cells while proviral levels remain stable in precursor T_N_, T_SCM_, and T_CM_ subsets (80, 134, 207), another possible strategy—to promote CD4+ T cell differentiation—has been posited. This effect could in theory be achieved by sequential polyclonal activation of the CD4+ T cell pool leading to “washout” of the reservoir (208). Modeling suggests this could have a modest impact on reservoir volume, which could be synergistic with anti-proliferative approaches (80). A limitation of this strategy is that, to our knowledge, no currently licensed drugs are known to have this effect in isolation, and it is unknown if pro-differentiation would impact the immunogenicity of reservoir cells or the effectiveness of the overall T cell immune responses.

## THE ROLE OF CELLULAR PROLIFERATION IN OBSERVED HIV CURES WITH STEM CELL TRANSPLANTATION

Seven PWH appear to have been cured of HIV following HSCT for concurrent hematologic malignancy (209). All but one of these individuals received cells from a homozygous delta32 CCR5-negative donor, rendering their CD4+ T cells resistant to *de novo* HIV infection (210). While the cell protection mechanism appears critical, the mechanism of cure is incompletely understood. The passenger hypothesis may partially explain this complex and multifactorial process. The first step in HSCT is partially myeloablative chemotherapy, which is associated with the elimination of all detectable white blood cells, including CD4+ T cells. HIV proviruses rapidly contract often to undetectable levels over the course of a week (210, 211). Infusion of donor stem cells repopulates the bone marrow and is followed by massive T cell activation and proliferation. White blood cell reconstitution may consist of both donor and host cells. The ratio of these populations is termed chimerism. If donor cells are not protected (CCR5−), HIV provirus levels usually rise during this period despite fully suppressive ART because HIV DNA is a passenger in reconstituting endogenous CD4+ T cells (211). Following successful transplantation, the rate of the reservoir decline correlates with donor cell chimerism, suggesting the donor graft may immunologically target remaining infected cells, accelerating negative selection dramatically (211). Therapeutic strategies that augment immune elimination of recovering HIV-infected cells during T cell reconstitution may therefore enhance the chance of prolonged remission (209).

Autologous stem cell transplantation is a less morbid procedure which is being considered as a method to achieve HIV cure without fully myeloablative conditioning regimens or the risk of donor-mediated graft versus host disease. CD4+ T cell recovery is invariably associated with an increase in HIV DNA levels, suggesting a key role for homeostatic proliferation (212). Experiments in macaques using SIV similarly demonstrate that activated CD4+ T cell proliferation following targeted lymphocyte depletion is associated with rapidly recovering SIV DNA to baseline levels (213), which is broadly in keeping with the passenger hypothesis. Given that CD4+ T cells are often highly activated during this proliferative recovery, therapies which augment anti-HIV immunity such as neutralizing antibodies or CAR T cells may be optimized if dosed soon after transplant (68, 174).

## CAVEATS AND LIMITATIONS OF THE PASSENGER HYPOTHESIS

As described above, the passenger hypothesis is insufficient to explain the entirety of reservoir dynamics and occurs in parallel with dynamic selection pressures which appear to wane with time on ART. There are several other possible issues not addressed by the passenger hypothesis. Myeloid-lineage cells may be part of the HIV reservoir, though it is an ongoing debate if they are a source of viral rebound following ATI (214). Macrophages do not proliferate and would not be targeted by therapies described above (214). Moreover, most studies and models cited above rely on HIV provirus sequence data from blood rather than lymphatic tissues where most of the reservoir exists, but studies show that blood and lymphoid provirus sequences are well admixed, and infected clones in blood can be found across several tissues (22, 45, 88, 96). Nevertheless, lymphatic tissue is undersampled to an even greater degree than blood. It is possible that trends observed in blood regarding clonality of reservoir cells are less pronounced in tissue. Attempts to more fully characterize the reservoir in human cadavers suggest clonal equivalence across multiple anatomic sites including spleen and other lymphatic sites, though profound undersampling remains a fundamental issue (96). Non-human primate studies allow sampling of multiple organs throughout the course of SIV infection and suggest that proliferative clones may exist in lymphatic tissues with roughly similar proportions to other clones (215). Yet, it remains possible that unobserved reservoir cells have important characteristics that deviate from the passenger hypothesis.

## SUMMARY

Our review suggests a vital role for uninfected CD4+ T cell physiology in explaining HIV reservoir dynamics with direct relevance for nearly all therapeutic strategies, reservoir measurement, and endpoint selection for clinical trials.

## References

[1] Siliciano RF, Siliciano JD, Blankson JN, Lai J, Rosenbloom DI, Laskey SB, Ho YC, Shan L, Wang J, Hosmane NN. 2013. Replication-competent noninduced proviruses in the latent reservoir increase barrier to HIV-1 cure. Cell 155:540–551. doi: 10.1016/j.cell.2013.09.020 24243014 10.1016/j.cell.2013.09.020 PMC3896327

[2] Siliciano JD, Kajdas J, Finzi D, Quinn TC, Chadwick K, Margolick JB, Kovacs C, Gange SJ, Siliciano RF. 2003. Long-term follow-up studies confirm the stability of the latent reservoir for HIV-1 in resting CD4+ T cells. Nat Med 9:727–728. doi:10.1038/nm880 12754504

[3] Finzi D, Hermankova M, Pierson T, Carruth LM, Buck C, Chaisson RE, Quinn TC, Chadwick K, Margolick J, Brookmeyer R, Gallant J, Markowitz M, Ho DD, Richman DD, Siliciano RF. 1997. Identification of a reservoir for HIV-1 in patients on highly active antiretroviral therapy. Science 278:1295–1300. doi:10.1126/science.278.5341.1295 9360927

[4] Falcinelli SD, Kilpatrick KW, Read J, Murtagh R, Allard B, Ghofrani S, Kirchherr J, James KS, Stuelke E, Baker C, Kuruc JD, Eron JJ, Hudgens MG, Gay CL, Margolis DM, Archin NM. 2021. Longitudinal dynamics of intact HIV proviral DNA and outgrowth virus frequencies in a cohort of individuals receiving antiretroviral therapy. J Infect Dis 224:92–100. doi:10.1093/infdis/jiaa718 33216132 PMC8253129

[5] Neidleman J, Luo X, Frouard J, Xie G, Hsiao F, Ma T, Morcilla V, Lee A, Telwatte S, Thomas R, Tamaki W, Wheeler B, Hoh R, Somsouk M, Vohra P, Milush J, James KS, Archin NM, Hunt PW, Deeks SG, Yukl SA, Palmer S, Greene WC, Roan NR. 2020. Phenotypic analysis of the unstimulated in vivo HIV CD4 T cell reservoir. Elife 9:e60933. doi:10.7554/eLife.60933 32990219 PMC7524554

[6] Castagna A, Muccini C, Galli L, Bigoloni A, Poli A, Spagnuolo V, Nozza S, Racca S, Galli A, Cinque P, Carini E, Lazzarin A. 2019. Analytical treatment interruption in chronic HIV-1 infection: time and magnitude of viral rebound in adults with 10 years of undetectable viral load and low HIV-DNA (APACHE study). J Antimicrob Chemother 74:2039–2046. doi:10.1093/jac/dkz138 31225610

[7] Zheng L, Tierney C, Bosch RJ. 2021. Analytical treatment interruption in HIV trials: statistical and study design considerations. Curr HIV/AIDS Rep 18:475–482. doi:10.1007/s11904-021-00569-8 34213731 PMC8251690

[8] Henrich TJ, Hatano H, Bacon O, Hogan LE, Rutishauser R, Hill A, Kearney MF, Anderson EM, Buchbinder SP, Cohen SE, et al.. 2017. HIV-1 persistence following extremely early initiation of antiretroviral therapy (ART) during acute HIV-1 infection: an observational study. PLoS Med 14:e1002417. doi:10.1371/journal.pmed.1002417 29112956 PMC5675377

[9] Deeks SG, Overbaugh J, Phillips A, Buchbinder S. 2015. HIV infection. Nat Rev Dis Primers 1:15035. doi:10.1038/nrdp.2015.35 27188527

[10] Moir S, Chun TW, Fauci AS. 2011. Pathogenic mechanisms of HIV disease. Annu Rev Pathol 6:223–248. doi:10.1146/annurev-pathol-011110-130254 21034222

[11] Gantner P, Buranapraditkun S, Pagliuzza A, Dufour C, Pardons M, Mitchell JL, Kroon E, Sacdalan C, Tulmethakaan N, Pinyakorn S, Robb ML, Phanuphak N, Ananworanich J, Hsu D, Vasan S, Trautmann L, Fromentin R, Chomont N. 2023. HIV rapidly targets a diverse pool of CD4^+^ T cells to establish productive and latent infections. Immunity 56:653–668. doi:10.1016/j.immuni.2023.01.030 36804957 PMC10023508

[12] Holte SE, Melvin AJ, Mullins JI, Tobin NH, Frenkel LM. 2006. Density-dependent decay in HIV-1 dynamics. J Acquir Immune Defic Syndr 41:266–276. doi:10.1097/01.qai.0000199233.69457.e4 16540927

[13] Perelson AS, Essunger P, Cao Y, Vesanen M, Hurley A, Saksela K, Markowitz M, Ho DD. 1997. Decay characteristics of HIV-1-infected compartments during combination therapy. Nature 387:188–191. doi:10.1038/387188a0 9144290

[14] Perelson AS, Neumann AU, Markowitz M, Leonard JM, Ho DD. 1996. HIV-1 dynamics in vivo: virion clearance rate, infected cell life-span, and viral generation time. Science 271:1582–1586. doi:10.1126/science.271.5255.1582 8599114

[15] Ho DD, Neumann AU, Perelson AS, Chen W, Leonard JM, Markowitz M. 1995. Rapid turnover of plasma virions and CD4 lymphocytes in HIV-1 infection. Nature 373:123–126. doi:10.1038/373123a0 7816094

[16] Cohn LB, Silva IT, Oliveira TY, Rosales RA, Parrish EH, Learn GH, Hahn BH, Czartoski JL, McElrath MJ, Lehmann C, Klein F, Caskey M, Walker BD, Siliciano JD, Siliciano RF, Jankovic M, Nussenzweig MC. 2015. HIV-1 integration landscape during latent and active infection. Cell 160:420–432. doi:10.1016/j.cell.2015.01.020 25635456 PMC4371550

[17] Mbonye U, Karn J. 2024. The cell biology of HIV-1 latency and rebound. Retrovirology (Auckl) 21:6. doi:10.1186/s12977-024-00639-w PMC10996279 38580979

[18] Siliciano RF, Greene WC. 2011. HIV latency. Cold Spring Harb Perspect Med 1:a007096. doi:10.1101/cshperspect.a007096 22229121 PMC3234450

[19] Zhou Y, Zhang H, Siliciano JD, Siliciano RF. 2005. Kinetics of human immunodeficiency virus type 1 decay following entry into resting CD4+ T cells. J Virol 79:2199–2210. doi:10.1128/JVI.79.4.2199-2210.2005 15681422 PMC546571

[20] Zack JA, Arrigo SJ, Weitsman SR, Go AS, Haislip A, Chen ISY. 1990. HIV-1 entry into quiescent primary lymphocytes: molecular analysis reveals a labile, latent viral structure. Cell 61:213–222. doi:10.1016/0092-8674(90)90802-L 2331748

[21] Pierson TC, Zhou Y, Kieffer TL, Ruff CT, Buck C, Siliciano RF. 2002. Molecular characterization of preintegration latency in human immunodeficiency virus type 1 infection. J Virol 76:8518–8531. doi:10.1128/jvi.76.17.8518-8513.2002 12163571 PMC136977

[22] von Stockenstrom S, Odevall L, Lee E, Sinclair E, Bacchetti P, Killian M, Epling L, Shao W, Hoh R, Ho T, Faria NR, Lemey P, Albert J, Hunt P, Loeb L, Pilcher C, Poole L, Hatano H, Somsouk M, Douek D, Boritz E, Deeks SG, Hecht FM, Palmer S. 2015. Longitudinal genetic characterization reveals that cell proliferation maintains a persistent HIV type 1 DNA pool during effective HIV therapy. J Infect Dis 212:596–607. doi:10.1093/infdis/jiv092 25712966 PMC4539896

[23] Josefsson L, King MS, Makitalo B, Brännström J, Shao W, Maldarelli F, Kearney MF, Hu WS, Chen J, Gaines H, Mellors JW, Albert J, Coffin JM, Palmer SE. 2011. Majority of CD4+ T cells from peripheral blood of HIV-1-infected individuals contain only one HIV DNA molecule. Proc Natl Acad Sci USA 108:11199–11204. doi:10.1073/pnas.1107729108 21690402 PMC3131354

[24] Josefsson L, Palmer S, Faria NR, Lemey P, Casazza J, Ambrozak D, Kearney M, Shao W, Kottilil S, Sneller M, Mellors J, Coffin JM, Maldarelli F. 2013. Single cell analysis of lymph node tissue from HIV-1 infected patients reveals that the majority of CD4+ T-cells contain one HIV-1 DNA molecule. PLoS Pathog 9:e1003432. doi:10.1371/journal.ppat.1003432 23818847 PMC3688524

[25] Cheng-Mayer C, Iannello P, Shaw K, Luciw PA, Levy JA. 1989. Differential effects of nef on HIV replication: implications for viral pathogenesis in the host. Science 246:1629–1632. doi:10.1126/science.2531920 2531920

[26] Planelles V, Bachelerie F, Jowett JB, Haislip A, Xie Y, Banooni P, Masuda T, Chen IS. 1995. Fate of the human immunodeficiency virus type 1 provirus in infected cells: a role for vpr. J Virol 69:5883–5889. doi:10.1128/JVI.69.9.5883-5889.1995 7637036 PMC189467

[27] Ramirez PW, DePaula-Silva AB, Szaniawski M, Barker E, Bosque A, Planelles V. 2015. HIV-1 Vpu utilizes both cullin-RING ligase (CRL) dependent and independent mechanisms to downmodulate host proteins. Retrovirology (Auckl) 12:65. doi:10.1186/s12977-015-0192-2 PMC4517359 26215564

[28] Planelles V, Barker E. 2010. Roles of Vpr and Vpx in modulating the virus-host cell relationship. Mol Aspects Med 31:398–406. doi:10.1016/j.mam.2010.05.002 20558198 PMC2967654

[29] Shen A, Siliciano JD, Pierson TC, Buck CB, Siliciano RF. 2000. Establishment of latent HIV-1 infection of resting CD4+ T lymphocytes does not require inactivation of Vpr. Virology (Auckl) 278:227–233. doi:10.1006/viro.2000.0650 11112497

[30] Omondi FH, Chandrarathna S, Mujib S, Brumme CJ, Jin SW, Sudderuddin H, Miller RL, Rahimi A, Laeyendecker O, Bonner P, Yue FY, Benko E, Kovacs CM, Brockman MA, Ostrowski M, Brumme ZL. 2019. HIV subtype and nef-mediated immune evasion function correlate with viral reservoir size in early-treated individuals. J Virol 93:e01832-18. doi:10.1128/JVI.01832-18 30602611 PMC6401425

[31] Romani B, Kamali Jamil R, Hamidi-Fard M, Rahimi P, Momen SB, Aghasadeghi MR, Allahbakhshi E. 2016. HIV-1 Vpr reactivates latent HIV-1 provirus by inducing depletion of class I HDACs on chromatin. Sci Rep 6:31924. doi:10.1038/srep31924 27550312 PMC4994036

[32] Wagner TA, McLaughlin S, Garg K, Cheung CYK, Larsen BB, Styrchak S, Huang HC, Edlefsen PT, Mullins JI, Frenkel LM. 2014. HIV latency. Proliferation of cells with HIV integrated into cancer genes contributes to persistent infection. Science 345:570–573. doi:10.1126/science.1256304 25011556 PMC4230336

[33] Maldarelli F, Wu X, Su L, Simonetti FR, Shao W, Hill S, Spindler J, Ferris AL, Mellors JW, Kearney MF, Coffin JM, Hughes SH. 2014. HIV latency. Specific HIV integration sites are linked to clonal expansion and persistence of infected cells. Science 345:179–183. doi:10.1126/science.1254194 24968937 PMC4262401

[34] Einkauf KB, Lee GQ, Gao C, Sharaf R, Sun X, Hua S, Chen SM, Jiang C, Lian X, Chowdhury FZ, Rosenberg ES, Chun TW, Li JZ, Yu XG, Lichterfeld M. 2019. Intact HIV-1 proviruses accumulate at distinct chromosomal positions during prolonged antiretroviral therapy. J Clin Invest 129:988–998. doi:10.1172/JCI124291 30688658 PMC6391088

[35] Antar AA, Jenike KM, Jang S, Rigau DN, Reeves DB, Hoh R, Krone MR, Keruly JC, Moore RD, Schiffer JT, Nonyane BA, Hecht FM, Deeks SG, Siliciano JD, Ho YC, Siliciano RF. 2020. Longitudinal study reveals HIV-1-infected CD4+ T cell dynamics during long-term antiretroviral therapy. J Clin Invest 130:3543–3559. doi:10.1172/JCI135953 32191639 PMC7324206

[36] Bruner KM, Murray AJ, Pollack RA, Soliman MG, Laskey SB, Capoferri AA, Lai J, Strain MC, Lada SM, Hoh R, Ho YC, Richman DD, Deeks SG, Siliciano JD, Siliciano RF. 2016. Defective proviruses rapidly accumulate during acute HIV-1 infection. Nat Med 22:1043–1049. doi:10.1038/nm.4156 27500724 PMC5014606

[37] Hiener B, Horsburgh BA, Eden JS, Barton K, Schlub TE, Lee E, Stockenstrom S, Odevall L, Milush JM, Liegler T, Sinclair E, Hoh R, Boritz EA, Douek D, Fromentin R, Chomont N, Deeks SG, Hecht FM, Palmer S. 2017. Identification of genetically intact HIV-1 proviruses in specific CD4. Cell Rep 21:813–822. doi:10.1016/j.celrep.2017.09.081 29045846 PMC5960642

[38] Bruner KM, Wang Z, Simonetti FR, Bender AM, Kwon KJ, Sengupta S, Fray EJ, Beg SA, Antar AAR, Jenike KM, et al.. 2019. A quantitative approach for measuring the reservoir of latent HIV-1 proviruses. Nature 566:120–125. doi:10.1038/s41586-019-0898-8 30700913 PMC6447073

[39] Levy CN, Hughes SM, Roychoudhury P, Reeves DB, Amstuz C, Zhu H, Huang M-L, Wei Y, Bull ME, Cassidy NAJ, McClure J, Frenkel LM, Stone M, Bakkour S, Wonderlich ER, Busch MP, Deeks SG, Schiffer JT, Coombs RW, Lehman DA, Jerome KR, Hladik F. 2021. A highly multiplexed droplet digital PCR assay to measure the intact HIV-1 proviral reservoir. Cell Rep Med 2:100243. doi:10.1016/j.xcrm.2021.100243 33948574 PMC8080125

[40] Horsburgh BA, Palmer S. 2018. Measuring HIV persistence on antiretroviral therapy. Adv Exp Med Biol 1075:265–284. doi:10.1007/978-981-13-0484-2_11 30030797

[41] Crooks AM, Bateson R, Cope AB, Dahl NP, Griggs MK, Kuruc JD, Gay CL, Eron JJ, Margolis DM, Bosch RJ, Archin NM. 2015. Precise quantitation of the latent HIV-1 reservoir: implications for eradication strategies. J Infect Dis 212:1361–1365. doi:10.1093/infdis/jiv218 25877550 PMC4601910

[42] Schiffer JT, Levy C, Hughes SM, Pandey U, Padullo M, Jerome KR, Zhu H, Puckett K, Helgeson E, Harrington RD, Hladik F. 2022. Stable HIV reservoir despite prolonged low-dose mycophenolate to limit CD4+ T-cell proliferation. Open Forum Infect Dis 9:ofac620. doi:10.1093/ofid/ofac620 36519118 PMC9745781

[43] Gaebler C, Falcinelli SD, Stoffel E, Read J, Murtagh R, Oliveira TY, Ramos V, Lorenzi JCC, Kirchherr J, James KS, Allard B, Baker C, Kuruc JD, Caskey M, Archin NM, Siliciano RF, Margolis DM, Nussenzweig MC. 2021. Sequence evaluation and comparative analysis of novel assays for intact proviral HIV-1 DNA. J Virol 95:e01986-20. doi:10.1128/JVI.01986-20 33361426 PMC8094944

[44] Shelton EM, Reeves DB, Bender Ignacio RA. 2020. Initiation of antiretroviral therapy during primary HIV infection: effects on the latent HIV reservoir, including on analytic treatment interruptions. AIDS Rev 23:28–39. doi:10.24875/AIDSRev.20000001 33105471 PMC7987773

[45] Wong JK, Yukl SA. 2016. Tissue reservoirs of HIV. Curr Opin HIV AIDS 11:362–370. doi:10.1097/COH.0000000000000293 27259045 PMC4928570

[46] Schiffer JT, Schiffer CA. 2018. To what extent can mathematical modeling inform the design of clinical trials? The example of safe dose reduction of tyrosine kinase inhibitors in responding patients with chronic myeloid leukemia. Haematologica 103:1756–1757. doi:10.3324/haematol.2018.201897 30381415 PMC6278970

[47] Reeves DB, Gaebler C, Oliveira TY, Peluso MJ, Schiffer JT, Cohn LB, Deeks SG, Nussenzweig MC. 2023. Impact of misclassified defective proviruses on HIV reservoir measurements. Nat Commun 14:4186. doi:10.1038/s41467-023-39837-z 37443365 PMC10345136

[48] Gaebler C, Lorenzi JCC, Oliveira TY, Nogueira L, Ramos V, Lu CL, Pai JA, Mendoza P, Jankovic M, Caskey M, Nussenzweig MC. 2019. Combination of quadruplex qPCR and next-generation sequencing for qualitative and quantitative analysis of the HIV-1 latent reservoir. J Exp Med 216:2253–2264. doi:10.1084/jem.20190896 31350309 PMC6781006

[49] Abdel-Mohsen M, Richman D, Siliciano RF, Nussenzweig MC, Howell BJ, Martinez-Picado J, Chomont N, Bar KJ, Yu XG, Lichterfeld M, et al.. 2020. Recommendations for measuring HIV reservoir size in cure-directed clinical trials. Nat Med 26:1339–1350. doi:10.1038/s41591-020-1022-1 32895573 PMC7703694

[50] White JA, Simonetti FR, Beg S, McMyn NF, Dai W, Bachmann N, Lai J, Ford WC, Bunch C, Jones JL, Ribeiro RM, Perelson AS, Siliciano JD, Siliciano RF. 2022. Complex decay dynamics of HIV virions, intact and defective proviruses, and 2LTR circles following initiation of antiretroviral therapy. Proc Natl Acad Sci USA 119:e2120326119. doi:10.1073/pnas.2120326119 35110411 PMC8833145

[51] McMyn NF, Varriale J, Fray EJ, Zitzmann C, MacLeod H, Lai J, Singhal A, Moskovljevic M, Garcia MA, Lopez BM, et al.. 2023. The latent reservoir of inducible, infectious HIV-1 does not decrease despite decades of antiretroviral therapy. J Clin Invest 133. doi:10.1172/JCI171554 PMC10471168 37463049

[52] Golob JL, Stern J, Holte S, Kitahata MM, Crane HM, Coombs RW, Goecker E, Woolfrey AE, Harrington RD. 2018. HIV DNA levels and decay in a cohort of 111 long-term virally suppressed patients. AIDS 32:2113–2118. doi:10.1097/QAD.0000000000001948 30005008 PMC6136948

[53] Bachmann N, von Siebenthal C, Vongrad V, Turk T, Neumann K, Beerenwinkel N, Bogojeska J, Fellay J, Roth V, Kok YL, et al.. 2019. Determinants of HIV-1 reservoir size and long-term dynamics during suppressive ART. Nat Commun 10. doi:10.1038/s41467-019-10884-9 PMC6642170 31324762

[54] Peluso MJ, Bacchetti P, Ritter KD, Beg S, Lai J, Martin JN, Hunt PW, Henrich TJ, Siliciano JD, Siliciano RF, Laird GM, Deeks SG. 2020. Differential decay of intact and defective proviral DNA in HIV-1-infected individuals on suppressive antiretroviral therapy. JCI Insight 5:e132997. doi:10.1172/jci.insight.132997 32045386 PMC7101154

[55] Reeves DB, Duke ER, Hughes SM, Prlic M, Hladik F, Schiffer JT. 2017. Anti-proliferative therapy for HIV cure: a compound interest approach. Sci Rep 7:4011. doi:10.1038/s41598-017-04160-3 28638104 PMC5479830

[56] Hill AL, Rosenbloom DIS, Fu F, Nowak MA, Siliciano RF. 2014. Predicting the outcomes of treatment to eradicate the latent reservoir for HIV-1. Proc Natl Acad Sci USA 111:13475–13480. doi:10.1073/pnas.1406663111 25097264 PMC4169952

[57] Pinkevych M, Cromer D, Tolstrup M, Grimm AJ, Cooper DA, Lewin SR, Søgaard OS, Rasmussen TA, Kent SJ, Kelleher AD, Davenport MP. 2015. HIV reactivation from latency after treatment interruption occurs on average every 5-8 days—implications for HIV remission. PLoS Pathog 11:e1005000. doi:10.1371/journal.ppat.1005000 26133551 PMC4489624

[58] Brignall R, Cauchy P, Bevington SL, Gorman B, Pisco AO, Bagnall J, Boddington C, Rowe W, England H, Rich K, Schmidt L, Dyer NP, Travis MA, Ott S, Jackson DA, Cockerill PN, Paszek P. 2017. Integration of kinase and calcium signaling at the level of chromatin underlies inducible gene activation in T cells. J Immunol 199:2652–2667. doi:10.4049/jimmunol.1602033 28904128 PMC5632840

[59] Shaw JP, Utz PJ, Durand DB, Toole JJ, Emmel EA, Crabtree GR. 1988. Identification of a putative regulator of early T cell activation genes. Science 241:202–205. doi:10.1126/science.3260404 3260404

[60] Pahl HL. 1999. Activators and target genes of Rel/NF-kappaB transcription factors. Oncogene 18:6853–6866. doi:10.1038/sj.onc.1203239 10602461

[61] Pinzone MR, VanBelzen DJ, Weissman S, Bertuccio MP, Cannon L, Venanzi-Rullo E, Migueles S, Jones RB, Mota T, Joseph SB, Groen K, Pasternak AO, Hwang W-T, Sherman B, Vourekas A, Nunnari G, O’Doherty U. 2019. Longitudinal HIV sequencing reveals reservoir expression leading to decay which is obscured by clonal expansion. Nat Commun 10:728. doi:10.1038/s41467-019-08431-7 30760706 PMC6374386

[62] Wiegand A, Spindler J, Hong FF, Shao W, Cyktor JC, Cillo AR, Halvas EK, Coffin JM, Mellors JW, Kearney MF. 2017. Single-cell analysis of HIV-1 transcriptional activity reveals expression of proviruses in expanded clones during ART. Proc Natl Acad Sci USA 114:E3659–E3668. doi:10.1073/pnas.1617961114 28416661 PMC5422779

[63] Moskovljevic M, Dragoni F, Board NL, Wu F, Lai J, Zhang H, White JR, Hoh R, Lynn K, Tebas P, Mounzer K, Deeks SG, Montaner LJ, Siliciano JD, Siliciano RF, Simonetti FR. 2024. Cognate antigen engagement induces HIV-1 expression in latently infected CD4^+^ T cells from people on long-term antiretroviral therapy. Immunity 57:2928–2944. doi:10.1016/j.immuni.2024.11.002 39612916 PMC11896817

[64] Cillo AR, Sobolewski MD, Bosch RJ, Fyne E, Piatak M, Coffin JM, Mellors JW. 2014. Quantification of HIV-1 latency reversal in resting CD4+ T cells from patients on suppressive antiretroviral therapy. Proc Natl Acad Sci USA 111:7078–7083. doi:10.1073/pnas.1402873111 24706775 PMC4024870

[65] Gálvez C, Grau-Expósito J, Urrea V, Clotet B, Falcó V, Buzón MJ, Martinez-Picado J. 2021. Atlas of the HIV-1 reservoir in peripheral CD4 T cells of individuals on successful antiretroviral therapy. MBio 12:e0307821. doi:10.1128/mBio.03078-21 34844430 PMC8630536

[66] Wu VH, Nordin JML, Nguyen S, Joy J, Mampe F, del Rio Estrada PM, Torres-Ruiz F, González-Navarro M, Luna-Villalobos YA, Ávila-Ríos S, Reyes-Terán G, Tebas P, Montaner LJ, Bar KJ, Vella LA, Betts MR. 2023. Profound phenotypic and epigenetic heterogeneity of the HIV-1-infected CD4+ T cell reservoir. Nat Immunol 24:359–370. doi:10.1038/s41590-022-01371-3 36536105 PMC9892009

[67] Pardons M, Baxter AE, Massanella M, Pagliuzza A, Fromentin R, Dufour C, Leyre L, Routy JP, Kaufmann DE, Chomont N. 2019. Single-cell characterization and quantification of translation-competent viral reservoirs in treated and untreated HIV infection. PLoS Pathog 15:e1007619. doi:10.1371/journal.ppat.1007619 30811499 PMC6411230

[68] Rust BJ, Kean LS, Colonna L, Brandenstein KE, Poole NH, Obenza W, Enstrom MR, Maldini CR, Ellis GI, Fennessey CM, Huang ML, Keele BF, Jerome KR, Riley JL, Kiem HP, Peterson CW. 2020. Robust expansion of HIV CAR T cells following antigen boosting in ART-suppressed nonhuman primates. Blood 136:1722–1734. doi:10.1182/blood.2020006372 32614969 PMC7544543

[69] Lim S-Y, Lee J, Osuna CE, Vikhe P, Schalk DR, Chen E, Fray E, Kumar M, Schultz-Darken N, Rakasz E, et al.. 2024. Induction of durable remission by dual immunotherapy in SHIV-infected ART-suppressed macaques. Science 383:1104–1111. doi:10.1126/science.adf7966 38422185 PMC11022498

[70] Wu HL, Busman-Sahay K, Weber WC, Waytashek CM, Boyle CD, Bateman KB, Reed JS, Hwang JM, Shriver-Munsch C, Swanson T, et al.. 2023. Allogeneic immunity clears latent virus following allogeneic stem cell transplantation in SIV-infected ART-suppressed macaques. Immunity 56:1649–1663. doi:10.1016/j.immuni.2023.04.019 37236188 PMC10524637

[71] Sun W, Gao C, Hartana CA, Osborn MR, Einkauf KB, Lian X, Bone B, Bonheur N, Chun TW, Rosenberg ES, Walker BD, Yu XG, Lichterfeld M. 2023. Phenotypic signatures of immune selection in HIV-1 reservoir cells. Nature 614:309–317. doi:10.1038/s41586-022-05538-8 36599977 PMC9908552

[72] Lian Xiaodong, Seiger KW, Parsons EM, Gao C, Sun W, Gladkov GT, Roseto IC, Einkauf KB, Osborn MR, Chevalier JM, Jiang C, Blackmer J, Carrington M, Rosenberg ES, Lederman MM, McMahon DK, Bosch RJ, Jacobson JM, Gandhi RT, Peluso MJ, Chun T-W, Deeks SG, Yu XG, Lichterfeld M. 2023. Progressive transformation of the HIV-1 reservoir cell profile over two decades of antiviral therapy. Cell Host & Microbe 31:83–96. doi:10.1016/j.chom.2022.12.002 36596305 PMC9839361

[73] Einkauf KB, Osborn MR, Gao C, Sun W, Sun X, Lian X, Parsons EM, Gladkov GT, Seiger KW, Blackmer JE, Jiang C, Yukl SA, Rosenberg ES, Yu XG, Lichterfeld M. 2022. Parallel analysis of transcription, integration, and sequence of single HIV-1 proviruses. Cell 185:266–282. doi:10.1016/j.cell.2021.12.011 35026153 PMC8809251

[74] Lian X, Gao C, Sun X, Jiang C, Einkauf KB, Seiger KW, Chevalier JM, Yuki Y, Martin M, Hoh R, Peluso MJ, Carrington M, Ruiz-Mateos E, Deeks SG, Rosenberg ES, Walker BD, Lichterfeld M, Yu XG. 2021. Signatures of immune selection in intact and defective proviruses distinguish HIV-1 elite controllers. Sci Transl Med 13:eabl4097. doi:10.1126/scitranslmed.abl4097 34910552 PMC9202005

[75] Thomas AS, Jones KL, Gandhi RT, McMahon DK, Cyktor JC, Chan D, Huang SH, Truong R, Bosque A, Macedo AB, Kovacs C, Benko E, Eron JJ, Bosch RJ, Lalama CM, Simmens S, Walker BD, Mellors JW, Jones RB. 2017. T-cell responses targeting HIV Nef uniquely correlate with infected cell frequencies after long-term antiretroviral therapy. PLoS Pathog 13:e1006629. doi:10.1371/journal.ppat.1006629 28931091 PMC5624641

[76] Takata H, Mitchell JL, Pacheco J, Pagliuzza A, Pinyakorn S, Buranapraditkun S, Sacdalan C, Leyre L, Nathanson S, Kakazu JC, Intasan J, Prueksakaew P, Chomchey N, Phanuphak N, de Souza M, Haddad EK, Rolland M, Tovanabutra S, Vasan S, Hsu DC, Chomont N, Trautmann L, RV254/SEARCH010, RV304/SEARCH013. 2023. An active HIV reservoir during ART is associated with maintenance of HIV-specific CD8^+^ T cell magnitude and short-lived differentiation status. Cell Host Microbe 31:1494–1506. doi:10.1016/j.chom.2023.08.012 37708852 PMC10564289

[77] Pollack RA, Jones RB, Pertea M, Bruner KM, Martin AR, Thomas AS, Capoferri AA, Beg SA, Huang SH, Karandish S, Hao H, Halper-Stromberg E, Yong PC, Kovacs C, Benko E, Siliciano RF, Ho YC. 2017. Defective HIV-1 proviruses are expressed and can be recognized by cytotoxic T lymphocytes, which shape the proviral landscape. Cell Host Microbe 21:494–506. doi:10.1016/j.chom.2017.03.008 28407485 PMC5433942

[78] Huang S-H, Ren Y, Thomas AS, Chan D, Mueller S, Ward AR, Patel S, Bollard CM, Cruz CR, Karandish S, Truong R, Macedo AB, Bosque A, Kovacs C, Benko E, Piechocka-Trocha A, Wong H, Jeng E, Nixon DF, Ho Y-C, Siliciano RF, Walker BD, Jones RB. 2018. Latent HIV reservoirs exhibit inherent resistance to elimination by CD8+ T cells. J Clin Invest 128:876–889. doi:10.1172/JCI97555 29355843 PMC5785246

[79] Reeves DB, Rigau DN, Romero A, Zhang H, Simonetti FR, Varriale J, Hoh R, Zhang L, Smith KN, Montaner LJ, Rubin LH, Gange SJ, Roan NR, Tien PC, Margolick JB, Peluso MJ, Deeks SG, Schiffer JT, Siliciano JD, Siliciano RF, Antar AAR. 2024 Mild HIV-specific selective forces overlaying natural CD4+ T cell dynamics explain the clonality and decay dynamics of HIV reservoir cells. HIV/AIDS. doi:10.1101/2024.02.13.24302704 PMC12694735 40987290

[80] Reeves DB, Bacchus-Souffan C, Fitch M, Abdel-Mohsen M, Hoh R, Ahn H, Stone M, Hecht F, Martin J, Deeks SG, Hellerstein MK, McCune JM, Schiffer JT, Hunt PW. 2023. Estimating the contribution of CD4 T cell subset proliferation and differentiation to HIV persistence. Nat Commun 14:6145. doi:10.1038/s41467-023-41521-1 37783718 PMC10545742

[81] Schröder ARW, Shinn P, Chen H, Berry C, Ecker JR, Bushman F. 2002. HIV-1 integration in the human genome favors active genes and local hotspots. Cell 110:521–529. doi:10.1016/S0092-8674(02)00864-4 12202041

[82] Nickle DC, Jensen MA, Shriner D, Brodie SJ, Frenkel LM, Mittler JE, Mullins JI. 2003. Evolutionary indicators of human immunodeficiency virus type 1 reservoirs and compartments. J Virol 77:5540–5546. doi:10.1128/jvi.77.9.5540-5546.2003 12692259 PMC153940

[83] Cao S, Slack SD, Levy CN, Hughes SM, Jiang Y, Yogodzinski C, Roychoudhury P, Jerome KR, Schiffer JT, Hladik F, Woodrow KA. 2019. Hybrid nanocarriers incorporating mechanistically distinct drugs for lymphatic CD4^+^ T cell activation and HIV-1 latency reversal. Sci Adv 5:eaav6322. doi:10.1126/sciadv.aav6322 30944862 PMC6436934

[84] Kearney MF, Spindler J, Shao W, Yu S, Anderson EM, O’Shea A, Rehm C, Poethke C, Kovacs N, Mellors JW, Coffin JM, Maldarelli F. 2014. Lack of detectable HIV-1 molecular evolution during suppressive antiretroviral therapy. PLoS Pathog 10:e1004010. doi:10.1371/journal.ppat.1004010 24651464 PMC3961343

[85] Rosenbloom DIS, Hill AL, Laskey SB, Siliciano RF. 2017. Re-evaluating evolution in the HIV reservoir. Nature 551:E6–E9. doi:10.1038/nature24634 29168805 PMC6103791

[86] Reeves DB, Duke ER, Wagner TA, Palmer SE, Spivak AM, Schiffer JT. 2018. A majority of HIV persistence during antiretroviral therapy is due to infected cell proliferation. Nat Commun 9:4811. doi:10.1038/s41467-018-06843-5 30446650 PMC6240116

[87] Brodin J, Zanini F, Thebo L, Lanz C, Bratt G, Neher RA, Albert J. 2016. Establishment and stability of the latent HIV-1 DNA reservoir. Elife 5:e18889. doi:10.7554/eLife.18889 27855060 PMC5201419

[88] McManus WR, Bale MJ, Spindler J, Wiegand A, Musick A, Patro SC, Sobolewski MD, Musick VK, Anderson EM, Cyktor JC, Halvas EK, Shao W, Wells D, Wu X, Keele BF, Milush JM, Hoh R, Mellors JW, Hughes SH, Deeks SG, Coffin JM, Kearney MF. 2019. HIV-1 in lymph nodes is maintained by cellular proliferation during antiretroviral therapy. J Clin Invest 129:4629–4642. doi:10.1172/JCI126714 31361603 PMC6819093

[89] Bozzi G, Simonetti FR, Watters SA, Anderson EM, Gouzoulis M, Kearney MF, Rote P, Lange C, Shao W, Gorelick R, Fullmer B, Kumar S, Wank S, Hewitt S, Kleiner DE, Hattori J, Bale MJ, Hill S, Bell J, Rehm C, Grossman Z, Yarchoan R, Uldrick T, Maldarelli F. 2019. No evidence of ongoing HIV replication or compartmentalization in tissues during combination antiretroviral therapy: Implications for HIV eradication. Sci Adv 5. doi:10.1126/sciadv.aav2045 PMC6760922 31579817

[90] Paryad-Zanjani S, Jagarapu A, Piovoso MJ, Zurakowski R. 2023. Ongoing HIV replication in lymph node sanctuary sites in treated individuals contributes to the total latent HIV at a very slow rate. J Theor Biol 575:111651. doi:10.1016/j.jtbi.2023.111651 37898364 PMC10680438

[91] Wagner TA, McKernan JL, Tobin NH, Tapia KA, Mullins JI, Frenkel LM. 2013. An increasing proportion of monotypic HIV-1 DNA sequences during antiretroviral treatment suggests proliferation of HIV-infected cells. J Virol 87:1770–1778. doi:10.1128/JVI.01985-12 23175380 PMC3554159

[92] Gantner P, Pagliuzza A, Pardons M, Ramgopal M, Routy J-P, Fromentin R, Chomont N. 2020. Single-cell TCR sequencing reveals phenotypically diverse clonally expanded cells harboring inducible HIV proviruses during ART. Nat Commun 11:4089. doi:10.1038/s41467-020-17898-8 32796830 PMC7427996

[93] Chomont N, El-Far M, Ancuta P, Trautmann L, Procopio FA, Yassine-Diab B, Boucher G, Boulassel M-R, Ghattas G, Brenchley JM, Schacker TW, Hill BJ, Douek DC, Routy J-P, Haddad EK, Sékaly R-P. 2009. HIV reservoir size and persistence are driven by T cell survival and homeostatic proliferation. Nat Med 15:893–900. doi:10.1038/nm.1972 19543283 PMC2859814

[94] Bull ME, Learn GH, McElhone S, Hitti J, Lockhart D, Holte S, Dragavon J, Coombs RW, Mullins JI, Frenkel LM. 2009. Monotypic human immunodeficiency virus type 1 genotypes across the uterine cervix and in blood suggest proliferation of cells with provirus. J Virol 83:6020–6028. doi:10.1128/JVI.02664-08 19339344 PMC2687376

[95] Boritz EA, Darko S, Swaszek L, Wolf G, Wells D, Wu X, Henry AR, Laboune F, Hu J, Ambrozak D, et al.. 2016. Multiple origins of virus persistence during natural control of HIV infection. Cell 166:1004–1015. doi:10.1016/j.cell.2016.06.039 27453467 PMC4983216

[96] Dufour C, Ruiz MJ, Pagliuzza A, Richard C, Shahid A, Fromentin R, Ponte R, Cattin A, Wiche Salinas TR, Salahuddin S, Sandstrom T, Schinkel SB, Costiniuk CT, Jenabian M-A, Ancuta P, Routy J-P, Cohen ÉA, Brumme ZL, Power C, Angel JB, Chomont N. 2023. Near full-length HIV sequencing in multiple tissues collected postmortem reveals shared clonal expansions across distinct reservoirs during ART. Cell Rep 42:113053. doi:10.1016/j.celrep.2023.113053 37676762

[97] Guo S, Luke BT, Henry AR, Darko S, Brandt LD, Su L, Sun D, Wells D, Joseph KW, Demirov D, Halvas EK, Douek DC, Wu X, Mellors JW, Hughes SH. 2022. HIV infected CD4+ T cell clones are more stable than uninfected clones during long-term antiretroviral therapy. PLoS Pathog 18:e1010726. doi:10.1371/journal.ppat.1010726 36044447 PMC9432747

[98] Ferreira RC, Prodger JL, Redd AD, Poon AFY. 2021. Quantifying the clonality and dynamics of the within-host HIV-1 latent reservoir. Virus Evol 7:veaa104. doi:10.1093/ve/veaa104 33505711 PMC7816690

[99] He F, Hubbell SP. 2011. Species-area relationships always overestimate extinction rates from habitat loss. Nature 473:368–371. doi:10.1038/nature09985 21593870

[100] Willis A. 2016. Extrapolating abundance curves has no predictive power for estimating microbial biodiversity. Proc Natl Acad Sci USA 113:E5096. doi:10.1073/pnas.1608281113 27512033 PMC5024625

[101] Orlitsky A, Suresh AT, Wu Y. 2016. Optimal prediction of the number of unseen species. Proc Natl Acad Sci USA 113:13283–13288. doi:10.1073/pnas.1607774113 27830649 PMC5127330

[102] Vibholm LK, Lorenzi JCC, Pai JA, Cohen YZ, Oliveira TY, Barton JP, Garcia Noceda M, Lu CL, Ablanedo-Terrazas Y, Del Rio Estrada PM, Reyes-Teran G, Tolstrup M, Denton PW, Damsgaard T, Søgaard OS, Nussenzweig MC. 2019. Characterization of intact proviruses in blood and lymph node from HIV-infected individuals undergoing analytical treatment interruption. J Virol 93:e01920-18. doi:10.1128/JVI.01920-18 30700598 PMC6450127

[103] Simonetti FR, Zhang H, Soroosh GP, Duan J, Rhodehouse K, Hill AL, Beg SA, McCormick K, Raymond HE, Nobles CL, Everett JK, Kwon KJ, White JA, Lai J, Margolick JB, Hoh R, Deeks SG, Bushman FD, Siliciano JD, Siliciano RF. 2021. Antigen-driven clonal selection shapes the persistence of HIV-1-infected CD4+ T cells in vivo. J Clin Invest 131:e145254. doi:10.1172/JCI145254 33301425 PMC7843227

[104] Hosmane NN, Kwon KJ, Bruner KM, Capoferri AA, Beg S, Rosenbloom DIS, Keele BF, Ho Y-C, Siliciano JD, Siliciano RF. 2017. Proliferation of latently infected CD4^+^ T cells carrying replication-competent HIV-1: Potential role in latent reservoir dynamics. J Exp Med 214:959–972. doi:10.1084/jem.20170193 28341641 PMC5379987

[105] Wang Z, Gurule EE, Brennan TP, Gerold JM, Kwon KJ, Hosmane NN, Kumar MR, Beg SA, Capoferri AA, Ray SC, Ho YC, Hill AL, Siliciano JD, Siliciano RF. 2018. Expanded cellular clones carrying replication-competent HIV-1 persist, wax, and wane. Proc Natl Acad Sci USA 115. doi:10.1073/pnas.1720665115 PMC5856552 29483265

[106] Simonetti FR, Sobolewski MD, Fyne E, Shao W, Spindler J, Hattori J, Anderson EM, Watters SA, Hill S, Wu X, et al.. 2016. Clonally expanded CD4+ T cells can produce infectious HIV-1 in vivo. Proc Natl Acad Sci USA 113:1883–1888. doi:10.1073/pnas.1522675113 26858442 PMC4763755

[107] Bui JK, Sobolewski MD, Keele BF, Spindler J, Musick A, Wiegand A, Luke BT, Shao W, Hughes SH, Coffin JM, Kearney MF, Mellors JW. 2017. Proviruses with identical sequences comprise a large fraction of the replication-competent HIV reservoir. PLoS Pathog 13:e1006283. doi:10.1371/journal.ppat.1006283 28328934 PMC5378418

[108] Cole B, Lambrechts L, Boyer Z, Noppe Y, De Scheerder M-A, Eden J-S, Vrancken B, Schlub TE, McLaughlin S, Frenkel LM, Palmer S, Vandekerckhove L. 2022. Extensive characterization of HIV-1 reservoirs reveals links to plasma viremia before and during analytical treatment interruption. Cell Rep 39:110739. doi:10.1016/j.celrep.2022.110739 35476994 PMC9745684

[109] De Scheerder M-A, Vrancken B, Dellicour S, Schlub T, Lee E, Shao W, Rutsaert S, Verhofstede C, Kerre T, Malfait T, Hemelsoet D, Coppens M, Dhondt A, De Looze D, Vermassen F, Lemey P, Palmer S, Vandekerckhove L. 2019. HIV rebound is predominantly fueled by genetically identical viral expansions from diverse reservoirs. Cell Host Microbe 26:347–358. doi:10.1016/j.chom.2019.08.003 31471273 PMC11021134

[110] White JA, Wu F, Yasin S, Moskovljevic M, Varriale J, Dragoni F, Camilo-Contreras A, Duan J, Zheng MY, Tadzong NF, et al.. 2023. Clonally expanded HIV-1 proviruses with 5′-leader defects can give rise to nonsuppressible residual viremia. J Clin Invest 133. doi:10.1172/JCI165245 PMC10014112 36602866

[111] Bailey JR, Sedaghat AR, Kieffer T, Brennan T, Lee PK, Wind-Rotolo M, Haggerty CM, Kamireddi AR, Liu Y, Lee J, Persaud D, Gallant JE, Cofrancesco J, Quinn TC, Wilke CO, Ray SC, Siliciano JD, Nettles RE, Siliciano RF. 2006. Residual human immunodeficiency virus type 1 viremia in some patients on antiretroviral therapy is dominated by a small number of invariant clones rarely found in circulating CD4 ^+^ T cells. J Virol 80:6441–6457. doi:10.1128/JVI.00591-06 16775332 PMC1488985

[112] Lu C-L, Pai JA, Nogueira L, Mendoza P, Gruell H, Oliveira TY, Barton J, Lorenzi JCC, Cohen YZ, Cohn LB, Klein F, Caskey M, Nussenzweig MC, Jankovic M. 2018. Relationship between intact HIV-1 proviruses in circulating CD4^+^ T cells and rebound viruses emerging during treatment interruption. Proc Natl Acad Sci USA 115:E11341–E11348. doi:10.1073/pnas.1813512115 30420517 PMC6275529

[113] Cohen YZ, Lorenzi JCC, Krassnig L, Barton JP, Burke L, Pai J, Lu C-L, Mendoza P, Oliveira TY, Sleckman C, Millard K, Butler AL, Dizon JP, Belblidia SA, Witmer-Pack M, Shimeliovich I, Gulick RM, Seaman MS, Jankovic M, Caskey M, Nussenzweig MC. 2018. Relationship between latent and rebound viruses in a clinical trial of anti–HIV-1 antibody 3BNC117. J Exp Med 215:2311–2324. doi:10.1084/jem.20180936 30072495 PMC6122972

[114] Lorenzi JCC, Cohen YZ, Cohn LB, Kreider EF, Barton JP, Learn GH, Oliveira T, Lavine CL, Horwitz JA, Settler A, Jankovic M, Seaman MS, Chakraborty AK, Hahn BH, Caskey M, Nussenzweig MC. 2016. Paired quantitative and qualitative assessment of the replication-competent HIV-1 reservoir and comparison with integrated proviral DNA. Proc Natl Acad Sci USA 113:E7908–E7916. doi:10.1073/pnas.1617789113 27872306 PMC5150408

[115] Halvas EK, Joseph KW, Brandt LD, Guo S, Sobolewski MD, Jacobs JL, Tumiotto C, Bui JK, Cyktor JC, Keele BF, Morse GD, Bale MJ, Shao W, Kearney MF, Coffin JM, Rausch JW, Wu X, Hughes SH, Mellors JW. 2020. HIV-1 viremia not suppressible by antiretroviral therapy can originate from large T cell clones producing infectious virus. J Clin Invest 130:5847–5857. doi:10.1172/JCI138099 33016926 PMC7598056

[116] Musick A, Spindler J, Boritz E, Pérez L, Crespo-Vélez D, Patro SC, Sobolewski MD, Bale MJ, Reid C, Keele BF, Capoferri A, Shao W, Wiegand A, Simonetti FR, Mellors JW, Hughes SH, Coffin JM, Maldarelli F, Kearney MF. 2019. HIV infected T cells can proliferate in vivo without inducing expression of the integrated provirus. Front Microbiol 10:2204. doi:10.3389/fmicb.2019.02204 31632364 PMC6781911

[117] Collora JA, Liu R, Pinto-Santini D, Ravindra N, Ganoza C, Lama JR, Alfaro R, Chiarella J, Spudich S, Mounzer K, Tebas P, Montaner LJ, van Dijk D, Duerr A, Ho Y-C. 2022. Single-cell multiomics reveals persistence of HIV-1 in expanded cytotoxic T cell clones. Immunity 55:1013–1031. doi:10.1016/j.immuni.2022.03.004 35320704 PMC9203927

[118] Clark IC, Mudvari P, Thaploo S, Smith S, Abu-Laban M, Hamouda M, Theberge M, Shah S, Ko SH, Pérez L, Bunis DG, Lee JS, Kilam D, Zakaria S, Choi S, Darko S, Henry AR, Wheeler MA, Hoh R, Butrus S, Deeks SG, Quintana FJ, Douek DC, Abate AR, Boritz EA. 2023. HIV silencing and cell survival signatures in infected T cell reservoirs. Nature 614:318–325. doi:10.1038/s41586-022-05556-6 36599978 PMC9908556

[119] Gandhi RT, Bosch RJ, Mar H, Laird GM, Halvas EK, Hovind L, Collier AC, Riddler SA, Martin A, Ritter K, McMahon DK, Eron JJ, Cyktor JC, Mellors JW, Team ACTGA. 2023. Varied patterns of decay of intact human immunodeficiency virus type 1 proviruses over 2 decades of antiretroviral therapy. J Infect Dis 227:1376–1380. doi:10.1093/infdis/jiad039 36763044 PMC10474937

[120] Mellors JW, Guo S, Naqvi A, Brandt LD, Su L, Sun Z, Joseph KW, Demirov D, Halvas EK, Butcher D, Scott B, Hamilton A, Heil M, Karim B, Wu X, Hughes SH. 2021. Insertional activation of STAT3 and LCK by HIV-1 proviruses in T cell lymphomas. Sci Adv 7:eabi8795. doi:10.1126/sciadv.abi8795 34644108 PMC8514100

[121] Gillis N, Dickey BL, Colin-Leitzinger C, Tang YH, Putney RM, Mesa TE, Yoder SJ, Suneja G, Spivak AM, Patel AB, Extermann M, Giuliano AR, Teng M, Kresovich J, Berglund A, Coghill AE. 2024. Clonal hematopoiesis in patients with human immunodeficiency virus and cancer. J Infect Dis 230:680–688. doi:10.1093/infdis/jiae212 38657098 PMC13031999

[122] Rocco JM, Zhou Y, Liu NS, Laidlaw E, Galindo F, Anderson MV, Rupert A, Lage SL, Ortega-Villa AM, Yu S, Lisco A, Manion M, Vassiliou GS, Dunbar CE, Sereti I. 2024. Clonal hematopoiesis in people with advanced HIV and associated inflammatory syndromes. JCI Insight 9:e174783. doi:10.1172/jci.insight.174783 38564303 PMC11141903

[123] McKinstry KK, Strutt TM, Swain SL. 2010. Regulation of CD4+ T-cell contraction during pathogen challenge. Immunol Rev 236:110–124. doi:10.1111/j.1600-065X.2010.00921.x 20636812 PMC2908916

[124] Marcus JL, Leyden WA, Alexeeff SE, Anderson AN, Hechter RC, Hu H, Lam JO, Towner WJ, Yuan Q, Horberg MA, Silverberg MJ. 2020. Comparison of overall and comorbidity-free life expectancy between insured adults with and without HIV infection, 2000-2016. JAMA Netw Open 3:e207954. doi:10.1001/jamanetworkopen.2020.7954 32539152 PMC7296391

[125] Horsburgh BA, Hiener B, Fisher K, Lee E, Morgan H, Eden J-S, von Stockenstrom S, Odevall L, Milush JM, Hoh R, Fromentin R, Chomont N, Hecht FM, Schlub TE, Deeks SG, Palmer S. 2022. Cellular activation, differentiation, and proliferation influence the dynamics of genetically intact proviruses over time. J Infect Dis 225:1168–1178. doi:10.1093/infdis/jiab291 34037766 PMC8974828

[126] Horsburgh BA, Lee E, Hiener B, Eden J-S, Schlub TE, von Stockenstrom S, Odevall L, Milush JM, Liegler T, Sinclair E, Hoh R, Boritz EA, Douek DC, Fromentin R, Chomont N, Deeks SG, Hecht FM, Palmer S. 2020. High levels of genetically intact HIV in HLA-DR+ memory T cells indicates their value for reservoir studies. AIDS 34:659–668. doi:10.1097/QAD.0000000000002465 31913161 PMC7071960

[127] Cho A, Gaebler C, Olveira T, Ramos V, Saad M, Lorenzi JCC, Gazumyan A, Moir S, Caskey M, Chun TW, Nussenzweig MC. 2022. Longitudinal clonal dynamics of HIV-1 latent reservoirs measured by combination quadruplex polymerase chain reaction and sequencing. Proc Natl Acad Sci USA 119:e2117630119. doi:10.1073/pnas.2117630119 35042816 PMC8794825

[128] Bosque A, Famiglietti M, Weyrich AS, Goulston C, Planelles V. 2011. Homeostatic proliferation fails to efficiently reactivate HIV-1 latently infected central memory CD4+ T cells. PLoS Pathog 7:e1002288. doi:10.1371/journal.ppat.1002288 21998586 PMC3188522

[129] De Boer RJ, Perelson AS. 2013. Quantifying T lymphocyte turnover. J Theor Biol 327:45–87. doi:10.1016/j.jtbi.2012.12.025 23313150 PMC3640348

[130] Antia R, Ganusov VV, Ahmed R. 2005. The role of models in understanding CD8+ T-cell memory. Nat Rev Immunol 5:101–111. doi:10.1038/nri1550 15662368

[131] Mendoza P, Jackson JR, Oliveira TY, Gaebler C, Ramos V, Caskey M, Jankovic M, Nussenzweig MC, Cohn LB. 2020. Antigen-responsive CD4+ T cell clones contribute to the HIV-1 latent reservoir. J Exp Med 217. doi:10.1084/jem.20200051 PMC7336300 32311008

[132] Faua C, Fafi-Kremer S, Gantner P. 2023. Antigen specificities of HIV-infected cells: A role in infection and persistence? J Virus Erad 9:100329. doi:10.1016/j.jve.2023.100329 37440870 PMC10334354

[133] Bacchus-Souffan C, Fitch M, Symons J, Abdel-Mohsen M, Reeves DB, Hoh R, Stone M, Hiatt J, Kim P, Chopra A, Ahn H, York VA, Cameron DL, Hecht FM, Martin JN, Yukl SA, Mallal S, Cameron PU, Deeks SG, Schiffer JT, Lewin SR, Hellerstein MK, McCune JM, Hunt PW. 2021. Relationship between CD4 T cell turnover, cellular differentiation and HIV persistence during ART. PLoS Pathog 17:e1009214. doi:10.1371/journal.ppat.1009214 33465157 PMC7846027

[134] Venanzi Rullo E, Pinzone MR, Cannon L, Weissman S, Ceccarelli M, Zurakowski R, Nunnari G, O’Doherty U. 2020. Persistence of an intact HIV reservoir in phenotypically naive T cells. JCI Insight 5:e133157. doi:10.1172/jci.insight.133157 33055422 PMC7605525

[135] Pinzone MR, Weissman S, Pasternak AO, Zurakowski R, Migueles S, O’Doherty U. 2021. Naive infection predicts reservoir diversity and is a formidable hurdle to HIV eradication. JCI Insight 6:e150794. doi:10.1172/jci.insight.150794 34228640 PMC8409977

[136] Wonderlich ER, Subramanian K, Cox B, Wiegand A, Lackman-Smith C, Bale MJ, Stone M, Hoh R, Kearney MF, Maldarelli F, Deeks SG, Busch MP, Ptak RG, Kulpa DA. 2019. Effector memory differentiation increases detection of replication-competent HIV-l in resting CD4+ T cells from virally suppressed individuals. PLoS Pathog 15:e1008074. doi:10.1371/journal.ppat.1008074 31609991 PMC6812841

[137] Brenchley JM, Schacker TW, Ruff LE, Price DA, Taylor JH, Beilman GJ, Nguyen PL, Khoruts A, Larson M, Haase AT, Douek DC. 2004. CD4+ T cell depletion during all stages of HIV disease occurs predominantly in the gastrointestinal tract. J Exp Med 200:749–759. doi:10.1084/jem.20040874 15365096 PMC2211962

[138] Coffin JM, Wells DW, Zerbato JM, Kuruc JD, Guo S, Luke BT, Eron JJ, Bale M, Spindler J, Simonetti FR, Hill S, Kearney MF, Maldarelli F, Wu X, Mellors JW, Hughes SH. 2019. Clones of infected cells arise early in HIV-infected individuals. JCI Insight 4. doi:10.1172/jci.insight.128432 PMC6629130 31217357

[139] Tettamanti Boshier FA, Reeves DB, Duke ER, Swan DA, Prlic M, Cardozo-Ojeda EF, Schiffer JT. 2022. Substantial uneven proliferation of CD4^+^ T cells during recovery from acute HIV infection is sufficient to explain the observed expanded clones in the HIV reservoir. J Virus Erad 8:100091. doi:10.1016/j.jve.2022.100091 36582473 PMC9792356

[140] Pankau MD, Reeves DB, Harkins E, Ronen K, Jaoko W, Mandaliya K, Graham SM, McClelland RS, Matsen Iv FA, Schiffer JT, Overbaugh J, Lehman DA. 2020. Dynamics of HIV DNA reservoir seeding in a cohort of superinfected Kenyan women. PLoS Pathog 16:e1008286. doi:10.1371/journal.ppat.1008286 32023326 PMC7028291

[141] Brooks K, Jones BR, Dilernia DA, Wilkins DJ, Claiborne DT, McInally S, Gilmour J, Kilembe W, Joy JB, Allen SA, Brumme ZL, Hunter E. 2020. HIV-1 variants are archived throughout infection and persist in the reservoir. PLoS Pathog 16:e1008378. doi:10.1371/journal.ppat.1008378 32492044 PMC7295247

[142] Kinloch NN, Shahid A, Dong W, Kirkby D, Jones BR, Beelen CJ, MacMillan D, Lee GQ, Mota TM, Sudderuddin H, Barad E, Harris M, Brumme CJ, Jones RB, Brockman MA, Joy JB, Brumme ZL. 2023. HIV reservoirs are dominated by genetically younger and clonally enriched proviruses. MBio 14:e0241723. doi:10.1128/mbio.02417-23 37971267 PMC10746175

[143] Brooks K, Omondi FH, Liang RH, Sudderuddin H, Jones BR, Joy JB, Brumme CJ, Hunter E, Brumme ZL. 2021. Proviral turnover during untreated HIV infection is dynamic and variable between hosts, impacting reservoir composition on ART. Front Microbiol 12:719153. doi:10.3389/fmicb.2021.719153 34489909 PMC8417368

[144] Wong M, Wei Y, Ho YC. 2023. Single-cell multiomic understanding of HIV-1 reservoir at epigenetic, transcriptional, and protein levels. Curr Opin HIV AIDS 18:246–256. doi:10.1097/COH.0000000000000809 37535039 PMC10442869

[145] Gandhi RT, Cyktor JC, Bosch RJ, Mar H, Laird GM, Martin A, Collier AC, Riddler SA, Macatangay BJ, Rinaldo CR, et al.. 2021. Selective decay of intact HIV-1 proviral DNA on antiretroviral therapy. J Infect Dis 223:225–233. doi:10.1093/infdis/jiaa532 32823274 PMC7857155

[146] Nettles RE, Kieffer TL, Kwon P, Monie D, Han Y, Parsons T, Cofrancesco J, Gallant JE, Quinn TC, Jackson B, Flexner C, Carson K, Ray S, Persaud D, Siliciano RF. 2005. Intermittent HIV-1 viremia (Blips) and drug resistance in patients receiving HAART. JAMA 293:817–829. doi:10.1001/jama.293.7.817 15713771

[147] Palmer S, Maldarelli F, Wiegand A, Bernstein B, Hanna GJ, Brun SC, Kempf DJ, Mellors JW, Coffin JM, King MS. 2008. Low-level viremia persists for at least 7 years in patients on suppressive antiretroviral therapy. Proc Natl Acad Sci USA 105:3879–3884. doi:10.1073/pnas.0800050105 18332425 PMC2268833

[148] Baxter AE, Niessl J, Fromentin R, Richard J, Porichis F, Charlebois R, Massanella M, Brassard N, Alsahafi N, Delgado GG, Routy JP, Walker BD, Finzi A, Chomont N, Kaufmann DE. 2016. Single-cell characterization of viral translation-competent reservoirs in hiv-infected individuals. Cell Host & Microbe 20:368–380. doi:10.1016/j.chom.2016.07.015 27545045 PMC5025389

[149] Kwon KJ, Timmons AE, Sengupta S, Simonetti FR, Zhang H, Hoh R, Deeks SG, Siliciano JD, Siliciano RF. 2020. Different human resting memory CD4^+^ T cell subsets show similar low inducibility of latent HIV-1 proviruses. Sci Transl Med 12:eaax6795. doi:10.1126/scitranslmed.aax6795 31996465 PMC7875249

[150] Siliciano JD, Siliciano RF. 2021. Low inducibility of latent human immunodeficiency virus type 1 proviruses as a major barrier to cure. J Infect Dis 223:S13–S21. doi:10.1093/infdis/jiaa649 PMC7883034 33586775

[151] Archin NM, Kirchherr JL, Sung JA, Clutton G, Sholtis K, Xu Y, Allard B, Stuelke E, Kashuba AD, Kuruc JD, Eron J, Gay CL, Goonetilleke N, Margolis DM. 2017. Interval dosing with the HDAC inhibitor vorinostat effectively reverses HIV latency. J Clin Invest 127:3126–3135. doi:10.1172/JCI92684 28714868 PMC5531421

[152] Warren JA, Zhou S, Xu Y, Moeser MJ, MacMillan DR, Council O, Kirchherr J, Sung JM, Roan NR, Adimora AA, Joseph S, Kuruc JD, Gay CL, Margolis DM, Archin N, Brumme ZL, Swanstrom R, Goonetilleke N. 2020. The HIV-1 latent reservoir is largely sensitive to circulating T cells. Elife 9:e57246. doi:10.7554/eLife.57246 33021198 PMC7593086

[153] Shan L, Deng K, Shroff NS, Durand CM, Rabi SA, Yang HC, Zhang H, Margolick JB, Blankson JN, Siliciano RF. 2012. Stimulation of HIV-1-specific cytolytic T lymphocytes facilitates elimination of latent viral reservoir after virus reactivation. Immunity 36:491–501. doi:10.1016/j.immuni.2012.01.014 22406268 PMC3501645

[154] Bertagnolli LN, Varriale J, Sweet S, Brockhurst J, Simonetti FR, White J, Beg S, Lynn K, Mounzer K, Frank I, Tebas P, Bar KJ, Montaner LJ, Siliciano RF, Siliciano JD. 2020. Autologous IgG antibodies block outgrowth of a substantial but variable fraction of viruses in the latent reservoir for HIV-1. Proc Natl Acad Sci USA 117:32066–32077. doi:10.1073/pnas.2020617117 33239444 PMC7749285

[155] Armani-Tourret M, Gao C, Hartana CA, Sun W, Carrere L, Vela L, Hochroth A, Bellefroid M, Sbrolla A, Shea K, Flynn T, Roseto I, Rassadkina Y, Lee C, Giguel F, Malhotra R, Bushman FD, Gandhi RT, Yu XG, Kuritzkes DR, Lichterfeld M. 2024. Selection of epigenetically privileged HIV-1 proviruses during treatment with panobinostat and interferon-α2a. Cell 187:1238–1254. doi:10.1016/j.cell.2024.01.037 38367616 PMC10903630

[156] Gasca-Capote C, Lian X, Gao C, Roseto IC, Jiménez-León MR, Gladkov G, Camacho-Sojo MI, Pérez-Gómez A, Gallego I, Lopez-Cortes LE, et al.. 2024. The HIV-1 reservoir landscape in persistent elite controllers and transient elite controllers. J Clin Invest 134. doi:10.1172/JCI174215 PMC11014653 38376918

[157] Jiang C, Lian X, Gao C, Sun X, Einkauf KB, Chevalier JM, Chen SMY, Hua S, Rhee B, Chang K, et al.. 2020. Distinct viral reservoirs in individuals with spontaneous control of HIV-1. Nature 585:261–267. doi:10.1038/s41586-020-2651-8 32848246 PMC7837306

[158] Mattapallil JJ, Douek DC, Hill B, Nishimura Y, Martin M, Roederer M. 2005. Massive infection and loss of memory CD4+ T cells in multiple tissues during acute SIV infection. Nature 434:1093–1097. doi:10.1038/nature03501 15793563

[159] Berkowitz RD, Beckerman KP, Schall TJ, McCune JM. 1998. CXCR4 and CCR5 expression delineates targets for HIV-1 disruption of T cell differentiation. J Immunol 161:3702–3710. doi:10.4049/jimmunol.161.7.3702 9759895

[160] Kufera JT, Armstrong C, Wu F, Singhal A, Zhang H, Lai J, Wilkins HN, Simonetti FR, Siliciano JD, Siliciano RF. 2024. CD4+ T cells with latent HIV-1 have reduced proliferative responses to T cell receptor stimulation. J Exp Med 221:e20231511. doi:10.1084/jem.20231511 38270554 PMC10818065

[161] Coffin JM, Bale MJ, Wells D, Guo S, Luke B, Zerbato JM, Sobolewski MD, Sia T, Shao W, Wu X, Maldarelli F, Kearney MF, Mellors JW, Hughes SH. 2021. Integration in oncogenes plays only a minor role in determining the in vivo distribution of HIV integration sites before or during suppressive antiretroviral therapy. PLoS Pathog 17:e1009141. doi:10.1371/journal.ppat.1009141 33826675 PMC8055010

[162] Cannon L, Fehrman S, Pinzone M, Weissman S, O’Doherty U. 2023. Machine learning bolsters evidence that D1, Nef, and Tat influence HIV reservoir dynamics. Pathog Immun 8:37–58. doi:10.20411/pai.v8i2.621 38292079 PMC10827039

[163] Luzuriaga K, Gay H, Ziemniak C, Sanborn KB, Somasundaran M, Rainwater-Lovett K, Mellors JW, Rosenbloom D, Persaud D. 2015. Viremic relapse after HIV-1 remission in a perinatally infected child. N Engl J Med 372:786–788. doi:10.1056/NEJMc1413931 25693029 PMC4440331

[164] Pinkevych M, Fennessey CM, Cromer D, Reid C, Trubey CM, Lifson JD, Keele BF, Davenport MP. 2019. Predictors of SIV recrudescence following antiretroviral treatment interruption. Elife 8:e49022. doi:10.7554/eLife.49022 31650954 PMC6917497

[165] Conway JM, Perelson AS. 2015. Post-treatment control of HIV infection. Proc Natl Acad Sci USA 112:5467–5472. doi:10.1073/pnas.1419162112 25870266 PMC4418889

[166] Hurst J, Hoffmann M, Pace M, Williams JP, Thornhill J, Hamlyn E, Meyerowitz J, Willberg C, Koelsch KK, Robinson N, Brown H, Fisher M, Kinloch S, Cooper DA, Schechter M, Tambussi G, Fidler S, Babiker A, Weber J, Kelleher AD, Phillips RE, Frater J. 2015. Immunological biomarkers predict HIV-1 viral rebound after treatment interruption. Nat Commun 6:8495. doi:10.1038/ncomms9495 26449164 PMC4633715

[167] Pereira Ribeiro S, Strongin Z, Soudeyns H, ten-Caten F, Ghneim K, Pacheco Sanchez G, Xavier de Medeiros G, Del Rio Estrada PM, Pelletier A-N, Hoang T, et al.. 2024. Dual blockade of IL-10 and PD-1 leads to control of SIV viral rebound following analytical treatment interruption. Nat Immunol 25:1900–1912. doi:10.1038/s41590-024-01952-4 39266691 PMC11436369

[168] Gunst JD, Gohil J, Li JZ, Bosch RJ, White, Catherine Seamon A, Chun T-W, Mothe B, Gittens K, Praiss L, De Scheerder M-A, et al.. 2025. Time to HIV viral rebound and frequency of post-treatment control after analytical interruption of antiretroviral therapy: an individual data-based meta-analysis of 24 prospective studies. Nat Commun 16. doi:10.1038/s41467-025-56116-1 PMC11751076 39837813

[169] Alexandre M, Prague M, Lhomme E, Lelièvre JD, Wittkop L, Richert L, Lévy Y, Thiébaut R. 2024. Definition of virological endpoints improving the design of HIV cure strategies using analytical antiretroviral treatment interruption. Clin Infect Dis 79:1447–1457. doi:10.1093/cid/ciae235 38819800

[170] Lau JSY, Cromer D, Pinkevych M, Lewin SR, Rasmussen TA, McMahon JH, Davenport MP. 2022. Balancing statistical power and risk in HIV cure clinical trial design. J Infect Dis 226:236–245. doi:10.1093/infdis/jiac032 35104873 PMC9400422

[171] Spivak AM, Planelles V. 2018. Novel latency reversal agents for HIV-1 cure. Annu Rev Med 69:421–436. doi:10.1146/annurev-med-052716-031710 29099677 PMC5892446

[172] Spivak AM, Bosque A, Balch AH, Smyth D, Martins L, Planelles V. 2015. Ex vivo bioactivity and HIV-1 latency reversal by ingenol dibenzoate and panobinostat in resting CD4(+) T cells from aviremic patients. Antimicrob Agents Chemother 59:5984–5991. doi:10.1128/AAC.01077-15 26169416 PMC4576101

[173] Armani-Tourret M, Bone B, Tan TS, Sun W, Bellefroid M, Struyve T, Louella M, Yu XG, Lichterfeld M. 2024. Immune targeting of HIV-1 reservoir cells: a path to elimination strategies and cure. Nat Rev Microbiol 22:328–344. doi:10.1038/s41579-024-01010-8 38337034 PMC11131351

[174] Zhen A, Peterson CW, Carrillo MA, Reddy SS, Youn CS, Lam BB, Chang NY, Martin HA, Rick JW, Kim J, Neel NC, Rezek VK, Kamata M, Chen ISY, Zack JA, Kiem H-P, Kitchen SG. 2017. Long-term persistence and function of hematopoietic stem cell-derived chimeric antigen receptor T cells in a nonhuman primate model of HIV/AIDS. PLoS Pathog 13:e1006753. doi:10.1371/journal.ppat.1006753 29284044 PMC5746250

[175] Gunst JD, Pahus MH, Rosás-Umbert M, Lu I-N, Benfield T, Nielsen H, Johansen IS, Mohey R, Østergaard L, Klastrup V, et al.. 2022. Early intervention with 3BNC117 and romidepsin at antiretroviral treatment initiation in people with HIV-1: a phase 1b/2a, randomized trial. Nat Med 28:2424–2435. doi:10.1038/s41591-022-02023-7 36253609 PMC10189540

[176] Rosás-Umbert M, Gunst JD, Pahus MH, Olesen R, Schleimann M, Denton PW, Ramos V, Ward A, Kinloch NN, Copertino DC, Escribà T, Llano A, Brumme ZL, Brad Jones R, Mothe B, Brander C, Fox J, Nussenzweig MC, Fidler S, Caskey M, Tolstrup M, Søgaard OS. 2022. Administration of broadly neutralizing anti-HIV-1 antibodies at ART initiation maintains long-term CD8+ T cell immunity. Nat Commun 13. doi:10.1038/s41467-022-34171-2 PMC9617872 36309514

[177] Yeh Y-HJ, Ho Y-C. 2021. Shock-and-kill versus block-and-lock: targeting the fluctuating and heterogeneous HIV-1 gene expression. Proc Natl Acad Sci USA 118. doi:10.1073/pnas.2103692118 PMC8072369 33758027

[178] Moranguinho I, Valente ST. 2020. Block-and-lock: new horizons for a cure for HIV-1. Viruses 12:1443. doi:10.3390/v12121443 33334019 PMC7765451

[179] Roychoudhury P, De Silva Feelixge HS, Pietz HL, Stone D, Jerome KR, Schiffer JT. 2016. Pharmacodynamics of anti-HIV gene therapy using viral vectors and targeted endonucleases. J Antimicrob Chemother 71:2089–2099. doi:10.1093/jac/dkw104 27090632 PMC4954920

[180] Schiffer JT, Aubert M, Weber ND, Mintzer E, Stone D, Jerome KR. 2012. Targeted DNA mutagenesis for the cure of chronic viral infections. J Virol 86:8920–8936. doi:10.1128/JVI.00052-12 22718830 PMC3416169

[181] Appel GB, Contreras G, Dooley MA, Ginzler EM, Isenberg D, Jayne D, Li LS, Mysler E, Sánchez-Guerrero J, Solomons N, Wofsy D, Group ALMS. 2009. Mycophenolate mofetil versus cyclophosphamide for induction treatment of lupus nephritis. J Am Soc Nephrol 20:1103–1112. doi:10.1681/ASN.2008101028 19369404 PMC2678035

[182] Chapuis AG, Paolo Rizzardi G, D’Agostino C, Attinger A, Knabenhans C, Fleury S, Acha-Orbea H, Pantaleo G. 2000. Effects of mycophenolic acid on human immunodeficiency virus infection in vitro and in vivo. Nat Med 6:762–768. doi:10.1038/77489 10888924

[183] García F, Plana M, Arnedo M, Brunet M, Castro P, Gil C, Vidal E, Millán O, López A, Martorell J, Fumero E, Miró JM, Alcamí J, Pumarola T, Gallart T, Gatell JM. 2004. Effect of mycophenolate mofetil on immune response and plasma and lymphatic tissue viral load during and after interruption of highly active antiretroviral therapy for patients with chronic HIV infection. JAIDS Journal of Acquired Immune Deficiency Syndromes 36:823–830. doi:10.1097/00126334-200407010-00009 15213566

[184] Katlama C, Lambert-Niclot S, Assoumou L, Papagno L, Lecardonnel F, Zoorob R, Tambussi G, Clotet B, Youle M, Achenbach CJ, Murphy RL, Calvez V, Costagliola D, Autran B. 2016. Treatment intensification followed by interleukin-7 reactivates HIV without reducing total HIV DNA. AIDS 30:221–230. doi:10.1097/QAD.0000000000000894 26684819

[185] Vandergeeten C, Fromentin R, DaFonseca S, Lawani MB, Sereti I, Lederman MM, Ramgopal M, Routy J-P, Sékaly R-P, Chomont N. 2013. Interleukin-7 promotes HIV persistence during antiretroviral therapy. Blood 121:4321–4329. doi:10.1182/blood-2012-11-465625 23589672 PMC3663425

[186] Sereti I, Dunham RM, Spritzler J, Aga E, Proschan MA, Medvik K, Battaglia CA, Landay AL, Pahwa S, Fischl MA, Asmuth DM, Tenorio AR, Altman JD, Fox L, Moir S, Malaspina A, Morre M, Buffet R, Silvestri G, Lederman MM, ACTG 5214 Study Team. 2009. IL-7 administration drives T cell-cycle entry and expansion in HIV-1 infection. Blood 113:6304–6314. doi:10.1182/blood-2008-10-186601 19380868 PMC2710926

[187] Stock PG, Barin B, Hatano H, Rogers RL, Roland ME, Lee T-H, Busch M, Deeks SG, for Solid Organ Transplantation in HIV Study Investigators. 2014. Reduction of HIV persistence following transplantation in HIV-infected kidney transplant recipients. Am J Transplant 14:1136–1141. doi:10.1111/ajt.12699 24698537 PMC4012326

[188] Varco-Merth BD, Brantley W, Marenco A, Duell DD, Fachko DN, Richardson B, Busman-Sahay K, Shao D, Flores W, Engelman K, Fukazawa Y, Wong SW, Skalsky RL, Smedley J, Axthelm MK, Lifson JD, Estes JD, Edlefsen PT, Picker LJ, Cameron CMA, Henrich TJ, Okoye AA. 2022. Rapamycin limits CD4+ T cell proliferation in simian immunodeficiency virus–infected rhesus macaques on antiretroviral therapy. J Clin Invest 132. doi:10.1172/JCI156063 PMC9106346 35316218

[189] Henrich TJ, Bosch RJ, Godfrey C, Mar H, Nair A, Keefer M, Fichtenbaum C, Moisi D, Clagett B, Buck AM, Deitchman AN, Aweeka F, Li JZ, Kuritzkes DR, Lederman MM, Hsue PY, Deeks SG, Team AA. 2024. Sirolimus reduces T cell cycling, immune checkpoint marker expression, and HIV-1 DNA in people with HIV. Cell Rep Med 5:101745. doi:10.1016/j.xcrm.2024.101745 39321793 PMC11513808

[190] Freeman ML, Clagett BM, Moisi D, Yeh E, Morris CD, Ryu A, Rodriguez B, Stein JH, Deeks SG, Currier JS, Hsue PY, Anthony DD, Calabrese LH, Ribaudo HJ, Lederman MM. 2022. Methotrexate inhibits T cell proliferation but not inflammatory cytokine expression to modulate immunity in people living with HIV. Front Immunol 13:924718. doi:10.3389/fimmu.2022.924718 35967371 PMC9374564

[191] Innis EA, Levinger C, Szaniawski MA, Williams E, Alcamí J, Bosque A, Schiffer JT, Coiras M, Spivak AM, Planelles V. 2021. Pharmacologic control of homeostatic and antigen-driven proliferation to target HIV-1 persistence. Biochem Pharmacol 194:114816. doi:10.1016/j.bcp.2021.114816 34715067 PMC8629953

[192] Kantarjian H, Shah NP, Hochhaus A, Cortes J, Shah S, Ayala M, Moiraghi B, Shen Z, Mayer J, Pasquini R, Nakamae H, Huguet F, Boqué C, Chuah C, Bleickardt E, Bradley-Garelik MB, Zhu C, Szatrowski T, Shapiro D, Baccarani M. 2010. Dasatinib versus imatinib in newly diagnosed chronic-phase chronic myeloid leukemia. N Engl J Med 362:2260–2270. doi:10.1056/NEJMoa1002315 20525995

[193] Fei F, Yu Y, Schmitt A, Rojewski MT, Chen B, Greiner J, Götz M, Guillaume P, Döhner H, Bunjes D, Schmitt M. 2008. Dasatinib exerts an immunosuppressive effect on CD8+ T cells specific for viral and leukemia antigens. Exp Hematol 36:1297–1308. doi:10.1016/j.exphem.2008.05.002 18619726

[194] Ambrosioni J, Coiras M, Alcamí J, Miró JM. 2017. Potential role of tyrosine kinase inhibitors during primary HIV-1 infection. Expert Rev Anti Infect Ther 15:421–423. doi:10.1080/14787210.2017.1308823 28322065

[195] Vigón L, Martínez-Román P, Rodríguez-Mora S, Torres M, Puertas MC, Mateos E, Salgado M, Navarro A, Sánchez-Conde M, Ambrosioni J, et al.. 2021. Provirus reactivation is impaired in HIV-1 infected individuals on treatment with dasatinib and antiretroviral therapy. Biochem Pharmacol 192:114666. doi:10.1016/j.bcp.2021.114666 34186065 PMC8478809

[196] Rodríguez-Mora S, Spivak AM, Szaniawski MA, López-Huertas MR, Alcamí J, Planelles V, Coiras M. 2019. Tyrosine kinase inhibition: a new perspective in the fight against HIV. Curr HIV/AIDS Rep 16:414–422. doi:10.1007/s11904-019-00462-5 31506864 PMC6814579

[197] Bermejo M, López-Huertas MR, García-Pérez J, Climent N, Descours B, Ambrosioni J, Mateos E, Rodríguez-Mora S, Rus-Bercial L, Benkirane M, Miró JM, Plana M, Alcamí J, Coiras M. 2016. Dasatinib inhibits HIV-1 replication through the interference of SAMHD1 phosphorylation in CD4+ T cells. Biochem Pharmacol 106:30–45. doi:10.1016/j.bcp.2016.02.002 26851491

[198] Reinwald M, Boch T, Hofmann WK, Buchheidt D. 2015. Risk of infectious complications in hemato-oncological patients treated with kinase inhibitors. Biomark Insights 10:55–68. doi:10.4137/BMI.S22430 PMC4841329 27127405

[199] O’Doherty U, Picado JM, Sáez-Cirión A. 2023. Highlights from the inaugural HIV reservoirs and immune control conference, October 1st–4th 2023, Malahide Ireland. PAI 8:161–169. doi:10.20411/pai.v8i1.653 PMC10753932 38155941

[200] Haas J, Singer T, Nowak K, Brust J, Göttmann U, Schnülle P, Krüger B, Krämer BK, Benck U. 2015. Renal transplantation in HIV-positive renal transplant recipients: experience at the Mannheim University Hospital. Transplant Proc 47:2791–2794. doi:10.1016/j.transproceed.2015.09.064 26680097

[201] Schilling WHK, Jittamala P, Watson JA, Boyd S, Luvira V, Siripoon T, Ngamprasertchai T, Batty EM, Cruz C, Callery JJ, et al.. 2024. Antiviral efficacy of molnupiravir versus ritonavir-boosted nirmatrelvir in patients with early symptomatic COVID-19 (PLATCOV): an open-label, phase 2, randomised, controlled, adaptive trial. Lancet Infect Dis 24:36–45. doi:10.1016/S1473-3099(23)00493-0 37778363 PMC7615401

[202] Slyker JA, Guthrie B, Pankau M, Tapia K, Wamalwa D, Benki-Nugent S, Ngugi E, Huang ML, Njuguna I, Langat A, John-Stewart G, Lehman D. 2021. Association between cytomegalovirus and epstein-barr virus viremia and human immunodeficiency virus DNA levels in the reservoir of Kenyan infants receiving antiretroviral therapy. J Infect Dis 223:1923–1927. doi:10.1093/infdis/jiaa640 33064809 PMC8176631

[203] Gianella S, Letendre S. 2016. Cytomegalovirus and HIV: a dangerous pas de deux. J Infect Dis 214 Suppl 2:S67–74. doi:10.1093/infdis/jiw217 27625433 PMC5021239

[204] Attaf M, Roider J, Malik A, Rius Rafael C, Dolton G, Prendergast AJ, Leslie A, Ndung’u T, Kløverpris HN, Sewell AK, Goulder PJ. 2020. Cytomegalovirus-mediated T cell receptor repertoire perturbation is present in early life. Front Immunol 11:1587. doi:10.3389/fimmu.2020.01587 33101265 PMC7554308

[205] Mohammadi A, Etemad B, Zhang X, Li Y, Bedwell GJ, Sharaf R, Kittilson A, Melberg M, Crain CR, Traunbauer AK, et al.. 2023. Viral and host mediators of non-suppressible HIV-1 viremia. Nat Med 29:3212–3223. doi:10.1038/s41591-023-02611-1 37957382 PMC10719098

[206] Arandjelovic P, Kim Y, Cooney JP, Preston SP, Doerflinger M, McMahon JH, Garner SE, Zerbato JM, Roche M, Tumpach C, Ong J, Sheerin D, Smyth GK, Anderson JL, Allison CC, Lewin SR, Pellegrini M. 2023. Venetoclax, alone and in combination with the BH3 mimetic S63845, depletes HIV-1 latently infected cells and delays rebound in humanized mice. Cell Reports Medicine 4:101178. doi:10.1016/j.xcrm.2023.101178 37652018 PMC10518630

[207] Jaafoura S, de Goër de Herve MG, Hernandez-Vargas EA, Hendel-Chavez H, Abdoh M, Mateo MC, Krzysiek R, Merad M, Seng R, Tardieu M, Delfraissy JF, Goujard C, Taoufik Y. 2014. Progressive contraction of the latent HIV reservoir around a core of less-differentiated CD4. Nat Commun 5:5407. doi:10.1038/ncomms6407 25382623 PMC4241984

[208] Grossman Z, Singh NJ, Simonetti FR, Lederman MM, Douek DC, Deeks SG, Kawabe T, Bocharov G, Meier-Schellersheim M, Alon H, Chomont N, Grossman Z, Sousa AE, Margolis L, Maldarelli F. 2020. “Rinse and replace”: boosting T cell turnover to reduce HIV-1 reservoirs. Trends Immunol 41:466–480. doi:10.1016/j.it.2020.04.003 32414695

[209] Cleary M, Ndhlovu LC, Sacha JB. 2025. Stem cell transplantation and allogeneic immunity: post treatment control or HIV cure? Curr Opin HIV AIDS 20:86–91. doi:10.1097/COH.0000000000000892 39484913 PMC11620935

[210] Sáez-Cirión A, Mamez A-C, Avettand-Fenoel V, Nabergoj M, Passaes C, Thoueille P, Decosterd L, Hentzien M, Perdomo-Celis F, Salgado M, Nijhuis M, Mélard A, Gardiennet E, Lorin V, Monceaux V, Chapel A, Gourvès M, Lechartier M, Mouquet H, Wensing A, Martinez-Picado J, Yerly S, Rougemont M, Calmy A. 2024. Sustained HIV remission after allogeneic hematopoietic stem cell transplantation with wild-type CCR5 donor cells. Nat Med 30:3544–3554. doi:10.1038/s41591-024-03277-z 39222660 PMC11645271

[211] Salgado M, Gálvez C, Nijhuis M, Kwon M, Cardozo-Ojeda EF, Badiola J, Gorman MJ, Huyveneers LEP, Urrea V, Bandera A, et al.. 2024. Dynamics of virological and immunological markers of HIV persistence after allogeneic haematopoietic stem-cell transplantation in the IciStem cohort: a prospective observational cohort study. Lancet HIV 11:e389–e405. doi:10.1016/S2352-3018(24)00090-0 38816141 PMC11417461

[212] Cardozo-Ojeda EF, Duke ER, Peterson CW, Reeves DB, Mayer BT, Kiem H-P, Schiffer JT. 2021. Thresholds for post-rebound SHIV control after CCR5 gene-edited autologous hematopoietic cell transplantation. Elife 10:e57646. doi:10.7554/eLife.57646 33432929 PMC7803377

[213] Varco-Merth B, Chaunzwa M, Duell DM, Marenco A, Goodwin W, Dannay R, Nekorchuk M, Shao D, Busman-Sahay K, Fennessey CM, et al.. 2024. Impact of alemtuzumab-mediated lymphocyte depletion on SIV reservoir establishment and persistence. PLoS Pathog 20:e1012496. doi:10.1371/journal.ppat.1012496 39173097 PMC11373844

[214] Veenhuis RT, Abreu CM, Costa PAG, Ferreira EA, Ratliff J, Pohlenz L, Shirk EN, Rubin LH, Blankson JN, Gama L, Clements JE. 2023. Monocyte-derived macrophages contain persistent latent HIV reservoirs. Nat Microbiol 8:833–844. doi:10.1038/s41564-023-01349-3 36973419 PMC10159852

[215] Mudd JC. 2024. Quantitative and qualitative distinctions between HIV-1 and SIV reservoirs: implications for hiv-1 cure-related studies. Viruses 16:514. doi:10.3390/v16040514 38675857 PMC11054464

